# Terpenoid Emissions of Two Mediterranean Woody Species in Response to Drought Stress

**DOI:** 10.3389/fpls.2018.01071

**Published:** 2018-07-23

**Authors:** Simon Haberstroh, Jürgen Kreuzwieser, Raquel Lobo-do-Vale, Maria C. Caldeira, Maren Dubbert, Christiane Werner

**Affiliations:** ^1^Ecosystem Physiology, University of Freiburg, Freiburg, Germany; ^2^Centro de Estudos Florestais, Instituto Superior de Agronomia, Universidade de Lisboa, Lisbon, Portugal

**Keywords:** BVOC, drought stress, adaptation, Mediterranean ecosystems, *Quercus suber*, *Cistus ladanifer*

## Abstract

Drought is a major environmental constrain affecting plant performance and survival, particularly in Mediterranean ecosystems. Terpenoids may play a protective role under these conditions, however, observations of drought effects on plant terpenoid emissions are controversial ranging from decreased emissions to unaffected or increased release of terpenoids. In the present study we investigated terpenoid emissions of cork oak (*Quercus suber*) and gum rockrose (*Cistus ladanifer*) in response to summer drought stress in 2017. Pre-dawn leaf water potential (Ψ_PD_) decreased from -0.64 to -1.72 MPa in *Q. suber* and from -1.69 to -4.05 MPa in *C. ladanifer*, indicating a transition from mild to severe drought along summer. Total terpenoid emissions decreased with drought, but differed significantly between species (*p* < 0.001) and in response to Ψ_PD_, air temperature and assimilation rates. *C. ladanifer* emitted a large variety of >75 compounds comprising monoterpenes, sesquiterpenes and even diterpenes, which strongly decreased from 1.37 ± 0.23 μg g^-1^h^-1^ to 0.40 ± 0.08 μg g^-1^h^-1^ (*p* < 0.001) in response to drought. Total emission rates were positively correlated to air temperature (*p* < 0.001). *C. ladanifer* behavior points toward terpenoid leaf storage depletion and reduced substrate availability for terpenoid synthesis with increasing drought, most likely accelerated by high air temperatures. *Q. suber* emitted mainly monoterpenes and emissions declined significantly from June (0.50 ± 0.08 μg g^-1^h^-1^) to August (0.29 ± 0.02 μg g^-1^h^-1^) (*p* < 0.01). Emission rates were weakly correlated with net assimilation rates (*R*^2^ = 0.19, *p* < 0.001), but did not respond strongly to Ψ_PD_ and air temperature. Early onset of drought in 2017 most likely reduced plant metabolism in *Q. suber*, resulting in diminished, but stable terpenoid fluxes. Calculation of standard emission factors (at 30°C) revealed contrasting emission patterns of decreasing, unaffected, or increasing fluxes of single terpenoid compounds. Unaffected or drought-enhanced emissions of compounds such as α-pinene, camphene or manoyl oxide may point toward a specific role of these terpenoids in abiotic stress adaptation. In conclusion, these results suggest a strong negative, but species- and compound-specific effect of severe drought on terpenoid fluxes in Mediterranean ecosystems.

## Introduction

Vegetation exerts a strong impact on atmospheric trace gasses, e.g., by buffering the effect of elevated CO_2_ through enhanced carbon sequestration, but inversely by emitting a diverse array of reactive hydrocarbons to the atmosphere. These biogenic volatile organic compounds (BVOC) are involved in a variety of functions in plants such as defense, reproduction or adaptation to stressful conditions (Kesselmeier and Staudt, 1999; Possell and Loreto, 2013). Terrestrial vegetation is estimated to emit around 1000 Tg carbon per year as BVOC (Guenther et al., 2012). Once released into the atmosphere BVOCs are highly reactive and exert a strong influence on atmospheric chemistry and air quality (Atkinson and Arey, 2003; Loreto et al., 2014), as they are involved in tropospheric ozone production, aerosol formation and ultimately influence climate (Holopainen and Gershenzon, 2010). BVOCs include a variety of chemical compounds such as terpenoids, alkanes, alkenes, alcohols, esters, carbonyls, or organic acids (Kesselmeier and Staudt, 1999; Dudareva et al., 2013), with terpenoids being the largest and most diverse cluster (Tholl, 2015). Terpenoids are organic substances all sharing a common C5 building block synthesized via the plastidic 2-C-methyl-D-erythritol 4-phosphate (MEP) pathway or the cytosolic mevalonic acid (MVA) pathway (Vickers et al., 2009; Tholl, 2015). Further transformation of C5 building blocks results in a large variety of compounds such as hemiterpenes (C5), monoterpenes (C10), sesquiterpenes (C15), diterpenes (C20), and terpenoids with even higher molecular mass (Vickers et al., 2009). Hemiterpenes, monoterpenes, and sesquiterpenes are considered to be volatile compounds as they have a high vapor pressure (Dudareva et al., 2006; Loreto et al., 2014). Diterpenes on the other hand, are either considered semi- or non-volatile (Niinemets, 2010; Loreto et al., 2014), but have recently been reported in terpenoid emissions of Mediterranean shrub species (Yáñez-Serrano et al., 2018). Especially emissions of the hemiterpene isoprene are thought to have a large influence on various protective mechanisms against abiotic and biotic stresses (Kesselmeier and Staudt, 1999; Loreto et al., 2014). In non-isoprene emitting plants, monoterpenes and sesquiterpenes are assumed to fulfill similar functions (Vickers et al., 2009; Loreto et al., 2014). These compounds act, for example, as membrane stabilizers, antioxidants or signal substances (Peñuelas et al., 2005; Vickers et al., 2009; Possell and Loreto, 2013).

While the response of BVOC emissions to abiotic factors such as temperature or light is well described (e.g., Kesselmeier and Staudt, 1999), results regarding their response to drought stress are more controversial (Staudt et al., 2002; Ormeno et al., 2007; Lluisà et al., 2016). Several studies suggest, that the intensity of stress appears to be the key predictor for emissions, as mild stress increases emissions, while they strongly decrease under severe drought (Ormeno et al., 2007; Lluisà et al., 2016). These emission patterns are often related to a decline in photosynthetic activity induced by prolonged drought (Lavoir et al., 2009). However, it is of high importance to comprehend the BVOC emission pattern of plants and ecosystems to drought stress, as the uncertainty of stress response strongly limits the reliability of models predicting BVOC emissions and, thus, projections of future emissions and impact on atmospheric chemistry (Niinemets, 2010; Guenther et al., 2012).

A region which is considered to contribute substantially to those uncertainties is the Mediterranean basin, where climate change impacts are already visible, such as prolonged drought periods and heat waves (Costa et al., 2010; Caldeira et al., 2015). Mediterranean ecosystems are characterized by pronounced summer drought and are, due to co-occurring high light intensities and air temperatures, strong BVOC emitters (Seco et al., 2011). Recent studies and models regarding climate change report an increasing risk of prolonged drought periods due to changed precipitation patterns and rising temperatures (Páscoa et al., 2017). Consequently, climate change will most likely influence BVOC emissions of Mediterranean ecosystems significantly. An excellent model system to study BVOC emission patterns in this regard are savannah type, man-made cork oak (*Quercus suber*) ecosystems, also called “montados” or “dehesas”. Given their large distribution, especially in the Iberian Peninsula (David et al., 2007), montado BVOC emissions may potentially affect regional atmospheric chemistry. In some areas, these socio-economically and ecologically important ecosystems are threatened by the invasion of shrubs such as gum rockrose (*Cistus ladanifer*), often as a result of land abandonment (Bugalho et al., 2011). While this native shrub itself has a high potential for BVOC emissions (Alías et al., 2012), it competes with *Q. suber* and *Q. ilex*, thereby reducing water and carbon fluxes, as well as resilience and resistance of trees (Rolo and Moreno, 2011; Caldeira et al., 2015). However, while we are only at the beginning of understanding the interaction between invasive species and native trees under drought (Rascher et al., 2011; Caldeira et al., 2015), even less is known, on how BVOC emissions of these different plant types respond to severe drought. To this end, we aim to shed new light onto the emission patterns of *Q. suber* and *C. ladanifer* under natural conditions in response to drought stress. We focus on terpenoids, since this BVOC class has been shown to play a vital role in plant stress responses (e.g., Dudareva et al., 2006). *Q. suber* is regarded as monoterpene emitter (Staudt et al., 2004, 2008; Pio et al., 2005; Bracho-Nunez et al., 2013) with a large intraspecific variability in emissions (Loreto et al., 2009). The lack of specialized storage organs for terpenoids indicates a high dependency of emissions on photosynthetic activity and light intensity (Loreto et al., 1996; Kesselmeier and Staudt, 1999). *C. ladanifer*, on the other hand, also emits monoterpenes (Pio et al., 1993), but possesses secretarial trichomes on its leaf surfaces, where terpenoids are accumulated (Gülz et al., 1996). This species has the potential to emit substantial amounts of monoterpenes, sesquiterpenes, and diterpenes (Yáñez-Serrano et al., 2018), which is in line with reported high terpenoid contents in essential oils of this species (Gomes et al., 2005; Verdeguer et al., 2012). Significant isoprene emissions have neither been detected from *Q. suber*, nor from *C. ladanifer* (Pio et al., 1993, 2005; Staudt et al., 2004). However, little is known on the influence of environmental drivers on terpenoid emissions of these species, particularly in response to prolonged summer drought. In this regard, we hypothesize that (i) the terpenoid emissions of *Q. suber* and *C. ladanifer* may rise with mild drought stress, but significantly decrease with severe plant water deficit, and that (ii) the emission patterns and emitted terpenoid compounds differ between the two investigated species.

## Materials and Methods

### Experimental Set-Up and Study Site

The effects of drought stress on terpenoid emissions were studied in a cork oak ecosystem partially invaded by the native shrub *C. ladanifer* in Vila Viçosa (Alentejo, 38° 47′ N, 7° 22′ W, 430 m a.s.l.), Portugal. The climate is characterized as typical Mediterranean with mild winters and a mean annual temperature of 15.9°C^1^ (Instituto Português do Mar e da Atmosfera [IPMA], 1981–2010). The bulk of the mean annual precipitation of 585 mm falls in winter, which leads to a distinct period of drought in summer (Caldeira et al., 2015). *Q. suber* is an evergreen tree belonging to the Eurasian subgenus *Cerris* and expressing a high intraspecific variability in plant traits (Manos et al., 1999; Loreto et al., 2009). Trees are adapted to the Mediterranean climate and withstand summer drought mainly by accessing deep water resources, hydraulic lift and stomatal control of transpiration (David et al., 2007; Grant et al., 2010). *C. ladanifer* is a woody semi-deciduous shrub belonging to the family Cistaceae which is well distributed in the Mediterranean Basin (Núñez-Olivera et al., 1996; Frazao et al., 2018). High growth rates and water-use-efficiency characterize this species (Correia et al., 1987; Werner et al., 1999; Correia and Ascensao, 2016). The density of *Q. suber* in this ecosystem is 160 ± 18.6 trees per ha. Shrubs form a dense understorey in monoculture (21,667 ± 2602 shrubs per ha), suppressing any other vegetation. Trees are approximately 6.6 ± 0.5 m high and on average 50 years old. The even aged *C. ladanifer* shrub layer reaches 2–3 m in height at an average age of 15 years. The soils are about 0.4 m deep with a high proportion of gravel, derived from schist and classified as haplic Leptosol (FAO, 2006). Terpenoid sampling and gas exchange measurements were conducted during three field campaigns in 2017 from 14 – 16 June, 11 – 13 July, and 2 – 4 August. Those dates usually represent three divergent phases of plant water status in the Mediterranean climate: (1) pre-drought period (2) onset of drought stress and (3) severe drought period (e.g., Otieno et al., 2006). All sampling days were characterized by stable weather conditions and clear skies. Measurements of meteorological conditions, water availability, sap flux density and leaf water potential were already started in May to characterize the meteorological and ecophysiological conditions prior to the terpenoid sampling.

### Meteorological Conditions and Water Availability

Meteorological parameters such as air temperature, relative humidity, precipitation and photosynthetically active photon flux density (PPFD) were retrieved continuously from a meteorological station installed on a scaffold tower and stored half-hourly on a data logger (DL2e, Delta-T Devices Ltd., Cambridge, United Kingdom). Vapour pressure deficit (VPD) was calculated from half-hourly values of air temperature and relative humidity. Further meteorological data was retrieved from a meteorological station nearby^1^ (Instituto Português do Mar e da Atmosfera [IPMA], 1981–2010). Volumetric soil water content from four different depths (0.1, 0.2, 0.3, and 0.4 m) was measured continuously with EC-10 probes (Decagon Devices, Pullman, WA, United States) in four profiles and stored half-hourly on a data logger (CR10X and AM16/32 multiplexer, Campbell Scientific, Logan, UT, United States).

### Ecophysiological Parameters

To determine the water status and overall physiological performance of the sampled plants, several ecophysiological parameters were measured during the field campaigns. Seven shrubs and nine trees were included to allow for a more robust identification of differences between species. Pre-dawn (Ψ_PD_) and midday (Ψ_MD_) leaf water potential measurements of *Q. suber* and *C. ladanifer* were conducted with a Scholander-type pressure chamber (PMS 1000, PMS Instruments, Corvalis, Oregon, OR, United States) between 3 and 6 am and 1 and 3 pm, respectively. Ψ_50_ for *Q. suber* and *C. ladanifer* was retrieved from literature (Quero et al., 2011; Pinto et al., 2012) and safety margins calculated as in Choat et al. (2012) as the difference of Ψ_MD_ and Ψ_50_. Ψ_50_ corresponds to the value where plants have already lost 50% of their hydraulic conductivity, and is considered to be a critical value, as surpassing this margin will likely result in persistent xylem damage and negative long-term effects (Choat et al., 2012). Sap flux density was measured continuously on site with Granier-type thermal dissipation probes (TDP30 sensors, Dynamax, Texas, United States) for seven trees as described in Caldeira et al. (2015). Thermal dissipation probes were installed radially at breast height with a north-west orientation to minimize the influence of external environmental factors on measurements. Due to the small diameter of the stems of *C*. *ladanifer*, sap flow of shrubs (*n* = 4) was measured via sap flow gauges (SGA13, Dynamax, Texas, United States) using the stem heat balance method of Sakuratani (1984). Measurements of terpenoids were not conducted on individuals with sap flow gauges, but on neighboring plants which were growing under the same conditions. Values for sap flow gauges and thermal dissipation probes were recorded every minute and stored as 30-min average on a data logger (CR1000 and AM16/32 multiplexer, Campbell Scientific, Logan, UT, United States). For determination of functional sap wood area of trees and shrubs, installation and protection of sensors see Caldeira et al. (2015). Gas exchange parameters such as net CO_2_ assimilation rate and stomatal conductance were recorded with a LI-6400XT portable photosynthesis system (LI-COR Inc., Nebraska, United States) with a light source and CO_2_ mixer. Measurements were conducted on sun exposed leaves of both species in the morning between 8 and 9 am and during midday between 1 and 2 pm. PPFD was set to 1200 μmol m^-2^s^-1^ for sun leaves, which is known to be saturating for photosynthesis (Tenhunen et al., 1985). CO_2_-concentration in the chamber was set to 400 ppm; relative humidity and leaf temperature followed ambient values. The flow through the system was set to 500 ml min^-1^. Due to the inaccessibility of leaves at the height of the sunlit tree canopy, large branches were cut and leaves immediately measured. Tests were previously performed, indicating stable gas exchange readings for about 2 min after cutting (Lobo-do-Vale et al., 2017, unpublished data). In rare cases of stomatal closure new branches were sampled. *C. ladanifer* and *Q. suber* leaves not filling the cuvette (6 cm^2^) completely were taken and measured for actual leaf area with a customary scanner (EPSON EXPRESSION 1680) and analyzed with the software WinSEEDLE (Regent Instruments Inc., Canada) in the laboratory. Afterwards, gas exchange was corrected for the obtained leaf area.

### Terpenoid Sampling

On each BVOC sampling day, terpenoids were measured on up to four different twigs of the same individual. In total, eight *Q. suber* and four *C. ladanifer* individuals were selected for measurements. Care was taken that always the same individuals were chosen. Selected twigs included visually healthy current or last year leaves 2 – 3 m above ground for *Q. suber* and 0.3 – 1 m above ground for *C. ladanifer*. Twigs from the lower part of the open tree canopies had to be selected for terpenoid sampling, due to constrains regarding the accessibility of the tree crown and to avoid condensation problems arising in fully sunlit enclosures in both species. Where necessary, twigs were shielded with neutral density meshes to avoid direct sunlight causing condensation due to enhanced transpiration and to assure a comparable sampling treatment. Terpenoids were collected using a dynamic enclosure system. Selected twigs were placed in custom-made enclosures (∼460 ml volume), which were made of chemically inert Nalophan foil (Bratschlauch, Toppits^®^, Minden, Germany) (Kessler et al., 2015) and perfluoroalkoxy (PFA) tubing (Swagelok, Karlsruhe, Germany). The outlets were connected via PFA tubing to air sampling pumps (210-1003MTX, SKC, Germany), to minimize terpenoid losses due to reactions and/or adsorption to enclosure walls and tubing. For emission measurement, twigs with 4–26 leaves were carefully placed into the enclosures, which were slightly, but not completely closed to allow non-treated, ambient air to enter the system. Prior to terpenoid sampling, enclosured twigs were flushed thoroughly for approximately 5 min to allow the leaves to acclimate to the new conditions. Subsequently, adsorbent tubes filled with polydimethylsiloxane (PDMS) foam (GERSTEL GmbH & Co. KG, Müllheim a.d. Ruhr, Germany) were installed between the outlet of the enclosure and the air sampling pumps for terpenoid trapping. During sampling, adsorbent tubes were covered with aluminum foil. The sampling time was set to 60 – 90 min at a flow rate of 200 ml min^-1^. In addition, controls with empty enclosures were installed approximately 2 m above ground and sampled concurrently to correct for ambient terpenoid concentrations. Immediately after sampling, twigs were cut and gas exchange was measured at a PPFD of 300 μmol m^-2^ s^-1^ to match the light conditions of terpenoid measurements under shaded conditions. Leaves were stored in a cooler bag for the determination of leaf area (see above) and dry leaf weight; adsorbent tubes were also stored cool before taking them to the laboratory, where they were kept at 4°C in Labco Exetainers (Labco Limited, Lampeter Ceredigion, United Kingdom) to avoid external influences until the analysis. For the determination of dry leaf weight, leaves were dried at 65°C for 48 h and weighed. The whole terpenoid sampling procedure was conducted between 8 am and 3 pm on each sampling day.

### Terpenoid Analysis

Terpenoids were analyzed on a gas chromatograph (GC, model 6890A, Agilent Technologies Böblingen, Germany) connected to a mass-selective detector (MSD, 5975C, Agilent Technologies Böblingen, Germany) and equipped with a thermodesorption/cold injection system (TDU-CIS, Gerstel, Germany). Sampling tubes were heated to 220°C, and, thermodesorbed volatiles channeled into the cold injection system where they were cryotrapped at -50°C; subsequently the cold injection system was heated to 240°C, releasing the volatiles onto the separation column (DB-5UI, Agilent Technologies Böblingen, Germany). Helium was used as a carrier gas at a flow of 1 ml min^-1^. The GC oven and MSD conditions as well as identification and quantification procedures are given in Kleiber et al. (2017). Briefly, the oven temperature began at 40°C, increasing at a rate of 6°C min^-1^ until 100°C were reached, thereafter the temperature ramp speeded up to 16°C min^-1^ until the oven was heated up to 230°C. The MSD was run at 70 eV at an ion source temperature of 230°C and a quadrupole temperature of 150°C. Retention index (RI) values were calculated using the tool of Lucero et al. (2009). As standards, the monoterpenes α-pinene, β-pinene, limonene and 1,8-cineole, the sesquiterpene caryophyllene and the diterpene ent-16-kaurene were chosen to quantify the final concentration of measured terpenoids. The mass spectra were analysed with the MassHunter Software (Agilent Technologies Böblingen, Germany). Measured terpenoid flux rates (*E*_m_) in μg g^-1^h^-1^ were calculated with equation 1:

$$E_{m}=\frac{\left(c_{o}-c_{i}\right)}{d_{w}*t}$$

where *c*_o_ (μg) is the terpenoid concentration in plant enclosures, *c*_i_ (μg) is the terpenoid background concentration in control samples, *d*_w_ (g) is the dry weight of leaves and *t* (*h*) is the sampling time. Terpenoids were grouped into monoterpenes (MT), oxygenated monoterpenes (MTO), sesquiterpenes (SQT), and diterpenes (DT). To account for the temperature dependence of emissions, standard emissions factors (*E*_s_) were calculated using equation 2 (Guenther et al., 1993) to standardize measured emission rates (*E*_m_) to a standard temperature (*T*_s_) of 30°C, by:

$$E_{m}=E_{s}\text{ exp }\left[\beta \left(T-T_{s}\right)\right]$$

where *T* (°C) is the ambient temperature during the terpenoid sampling, *T*_s_ is the standard temperature (30°C) and β (°C^-1^) is an empirical temperature coefficient varying for different terpenoids. *E*_s_ (μg C g^-1^ h^-1^) were calculated for all terpenoid compound groups and single compounds the same way for both species.

### Statistical Analysis

To identify significant differences in terpenoid emission rates, standard emission factors and ecophysiological parameters between species and over time, two-way repeated measure analysis of variance (RMANOVA) were performed and *post hoc* Tukey’s test applied when statistical differences were found. If the assumptions for ANOVA were not met (normal distribution, equality of variances), the data was transformed. Data was tested for normal distribution with the Shapiro–Wilks test. Terpenoid emission rates, standard emission factors, leaf water potential, sap flux density and gas exchange data were either log- or square root transformed. Sap flux density was fitted with a non-parametric smoothing kernel regression for plotting purposes. To test for correlations of environmental factors with terpenoid emissions, linear and exponential regressions were performed. Heat maps were created using the packages ‘MetaboAnalystR’ and ‘pheatmap’ in R. ‘MetaboAnalystR’ was used to log-transform and cluster emission data (method: ‘complete’) to identify similar emission patterns over time for single terpenoid compounds. The package ‘pheatmap’ allowed to produce publication ready heat maps. For statistical analysis and plots of terpenoid emissions only parameters of the measured plant individuals were used. Graphical plots except heat maps were created with SigmaPlot (version 14, Systat, United States). Statistical analysis and heat maps were conducted with the statistical software R (version 3.3.1 for Windows 10).

## Results

### Meteorological Conditions and Plant Water Status

**FIGURE 1:**
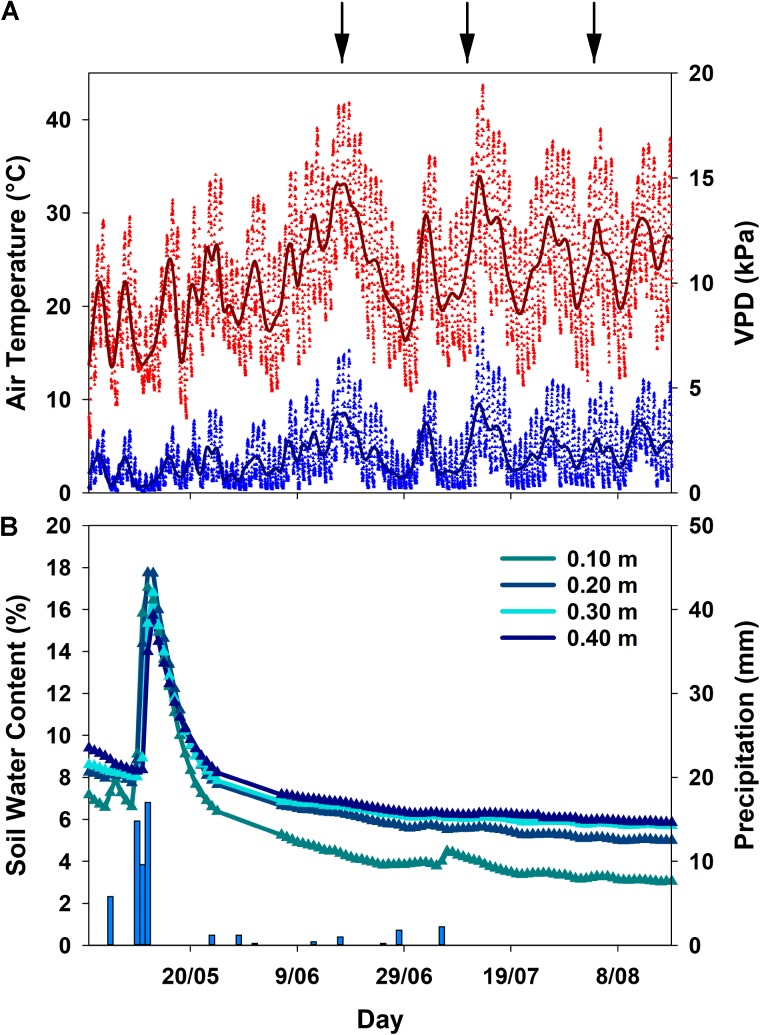
Smoothing mean for air temperature and vapor pressure deficit (VPD) **(A)**, mean daily soil water content (*n* = 4 per depth) and total precipitation **(B)** in Vila Viçosa during the sampling period. Symbols indicate values measured half-hourly. Due to sensor failure, the period between 25 May and 6 June represents an overall trend in soil water content, but not actually measured values. Arrows indicate the sampling campaigns.

Meteorological conditions in spring and summer 2017 were characterized by an early onset of drought due to rapidly rising air temperatures and only minor precipitation events (**Figure 1**). In general, all three sampling dates fell into heat waves with air temperatures and VPD reaching maximum values of 40.9°C and 6.2 kPa in June, 43.7°C and 7.9 kPa in July and 39.0°C and 5.3 kPa in August, respectively (**Figure 1A**). The last significant rainfall event occurred in May, thereafter soil water content declined, succeeded by only minor (<5 mm) precipitation, one occurring shortly before the second campaign in July as a short, intense convective event (**Figure 1B**). Therefore, cumulative precipitation from June to August was only 5.2 mm. The bulk of precipitation in the hydrological year fell in winter from October to February (325 mm), followed by 141 mm of rainfall from March to May prior to the terpenoid measurements. Overall precipitation of the hydrological year was 481 mm which is below the long-term average^2^ of 585 mm (Instituto Português do Mar e da Atmosfera [IPMA], 1981–2010).

**FIGURE 2:**
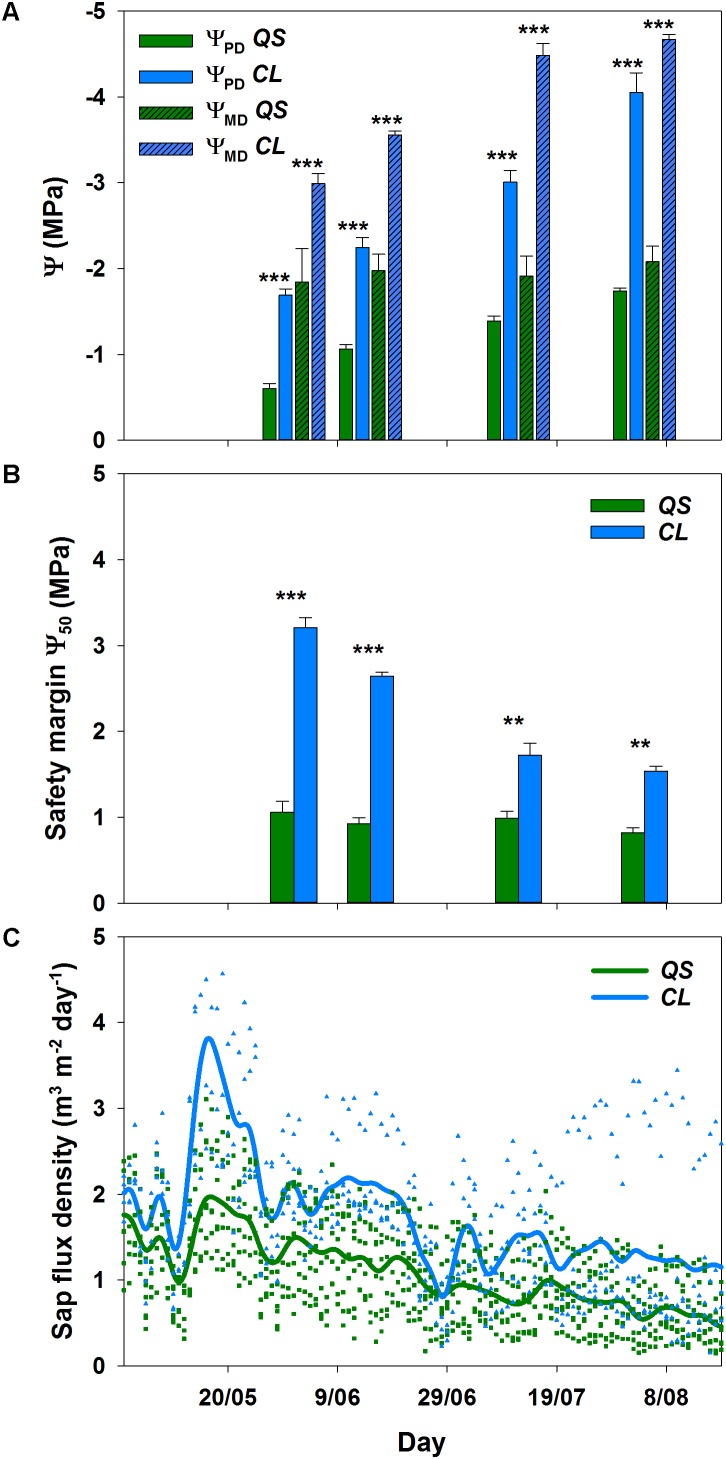
Pre-dawn (Ψ_PD_) and midday leaf water potential (Ψ_MD_) for *Quercus suber* (*QS*, *n* = 9) and *Cistus ladanifer* (*CL*, *n* = 7) with standard error **(A)**. Safety margins **(B)** were calculated according to Choat et al. (2012). Sap flux density for *Q. suber* (*QS*, *n* = 7) and *C. ladanifer* (*CL*, *n* = 4) was fitted as non-parametric kernel-regression **(C)**. Symbols are the values measured for each individual. Statistical differences (RMANOVA) between species are indicated by asterisks over bars at a significance level of ^∗^*p* < 0.05, ^∗∗^*p* < 0.01, ^∗∗∗^*p* < 0.001.

The decline in soil water content from mid-May onwards was visible in all soil depths (**Figure 1B**), and clearly reflected onto the plant water status of both species (**Figure 2**): a significant decrease (*p* < 0.001) in pre-dawn water potentials (Ψ_PD_) and sap flux density (*p* < 0.01) was evident for both species from the beginning to the end of the experiment. However, clear interspecific differences were detected in response to plant water deficit. *C. ladanifer* was able to endure lower Ψ_PD_ and Ψ_MD_, which strongly declined from June to August (from -1.69 MPa to -4.05 MPa and from -2.99 MPa to -4.67 MPa, respectively). Subsequently, safety margins at Ψ_50_, i.e., the margin to Ψ when 50% xylem cavitation occurs, diminished by more than half. Still *C. ladanifer* maintained a significantly higher safety margin in August, compared to *Q. suber* (*p* < 0.01). The deep-rooted oak on the other hand, maintained much higher water potentials through the entire drought period. While Ψ_PD_ responded to declining water resources and approached values of Ψ_MD_ (**Figures 2A,B**), midday water potentials never declined below -2 MPa and both, Ψ_MD_ and safety margins (0.95 ± 0.04 MPa) maintained stable over time. Such isohydric behavior came at the expense of lower sap flux density, which was markedly reduced in *Q. suber* over time (**Figure 2C**). In contrast, sap flux density was significantly higher in *C. ladanifer* throughout the measurement period (*p* < 0.001). Noticeably, sap flux density rapidly declined in both species from June onwards after a peak corresponding to the last strong rainfall in the middle of May. This indicates an early onset of drought in June with progressive development until August.

### Gas Exchange and Total Terpenoid Emissions

**FIGURE 3:**
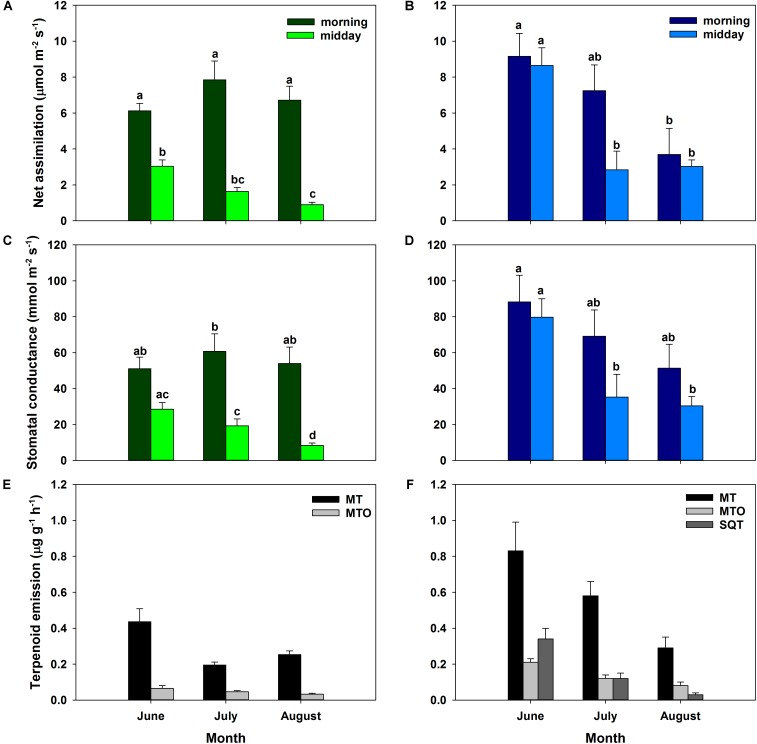
Assimilation rates and stomatal conductance for sun leaves for *Q. suber* (left, *n* = 9, **A,C**) and *C. ladanifer* (right, *n* = 7, **B,D**) with standard error for June, July, and August field campaigns. Letters indicate statistical differences (RMANOVA) of assimilation rates and stomatal conductance between sampling periods at a significance level of *p* < 0.05 for each species separately. Average measured terpenoid emissions of *Q. suber* (left, *n* = 6 for June, *n* = 8 for July and August, **E**) and *C. ladanifer* (right, *n* = 3 for June, *n* = 4 for July and August, **F**) with standard error. Emissions are grouped into monoterpenes (MT), oxygenated monoterpenes (MTO), and sesquiterpenes (SQT).

Carbon assimilation rates of *Q. suber* and *C. ladanifer* sun leaves illustrated two different patterns (**Figures 3A,B**). Net assimilation rates of *C. ladanifer* decreased from 9.16 ± 1.28 to 3.69 ± 1.45 μmol m^-2^ s^-1^ from June to August in the morning period (**Figure 3B**), as VPD increased and water availability declined. Stomatal conductance declined concomitantly, though not being as strongly reduced as net assimilation rates in August compared to June (**Figure 3D**). In June, *Q. suber* sun leaves showed already reduced assimilation of 6.12 ± 0.42 μmol m^-2^ s^-1^ compared to *C. ladanifer* (**Figure 3A**). However, *Q. suber* was able to maintain stable net assimilation rates over time, resulting in higher rates in August (6.71 ± 0.78 μmol m^-2^ s^-1^) at lower stomatal conductance, compared to *C. ladanifer* (**Figures 3C,D**). Especially for *Q. suber*, a midday depression of carbon assimilation was evident, responding to rising VPD and stomatal closure. Hence, highest net assimilation rates were measured in the morning period.

Similarly, to assimilation rates and stomatal conductance, total measured terpenoid emissions differed between species. Emissions of *Q. suber* were in the range of 0.24 – 0.50 μg g^-1^h^-1^ and decreased significantly from June to July (*p* < 0.01). Thereafter, a slight, but not significant increase in emissions occurred (**Figure 3E**). In total, 14 monoterpenes (MT), 10 oxygenated monoterpenes (MTO), and 8 sesquiterpenes (SQT) could be identified in *Q. suber*. SQT emissions decreased from 1.50 × 10^-3^ ± 5.50 × 10^-4^ μg g^-1^h^-1^ in June to 8.84 × 10^-5^ ± 2.30 × 10^-5^ μg g^-1^h^-1^ in August (data not shown). Overall emissions of *C. ladanifer* were in the range of 0.40 – 1.37 μg g^-1^h^-1^ and were not only significantly higher than those of *Q. suber* (*p* < 0.001) but also more variable and diverse. Emissions decreased consistently from June to August, which was most pronounced for SQT (**Figure 3F**). In August, terpenoid emissions reached a significantly lower level (*p* < 0.01) than in previous months, comparable to the emissions of *Q. suber*. Overall, emissions comprised 14 MTs, 19 MTOs, 37 SQTs, and even 4 diterpenes (DT). Measured emissions of the DTs cembrene, manoyl oxide, verticillol and ent-16-kaurene were low at rates of 7.92 × 10^-5^ – 13.97 × 10^-5^ μg g^-1^h^-1^, but the exclusive presence of these species in terpenoid emissions is noticeable.

### Correlations of Terpenoid Emission With Environmental Factors

**FIGURE 4:**
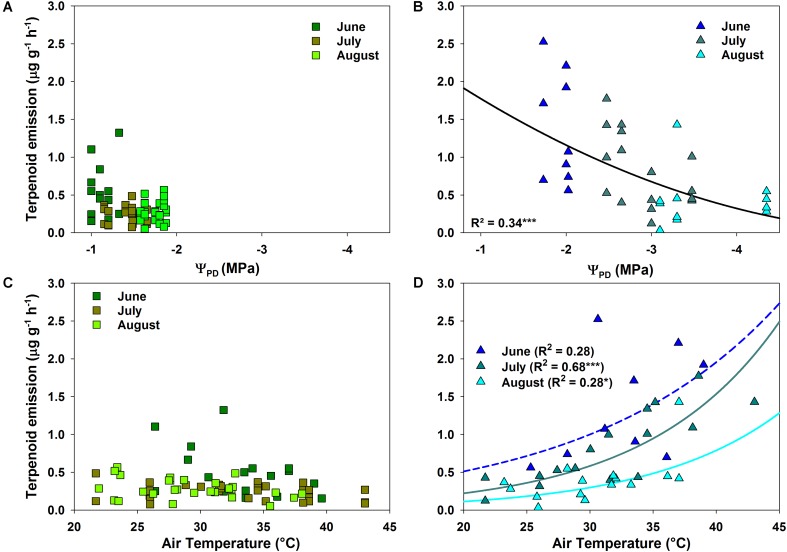
Response of measured terpenoid emission rates to pre-dawn leaf water potential (Ψ_PD_) and air temperature for *Q. suber* **(A,C)** and *C. ladanifer* **(B,D)**. Significant trends (linear and exponential regressions) are illustrated by solid lines at a significance levels of ^∗^*p* < 0.05, ^∗∗^*p* < 0.01, ^∗∗∗^*p* < 0.001.

**FIGURE 5:**
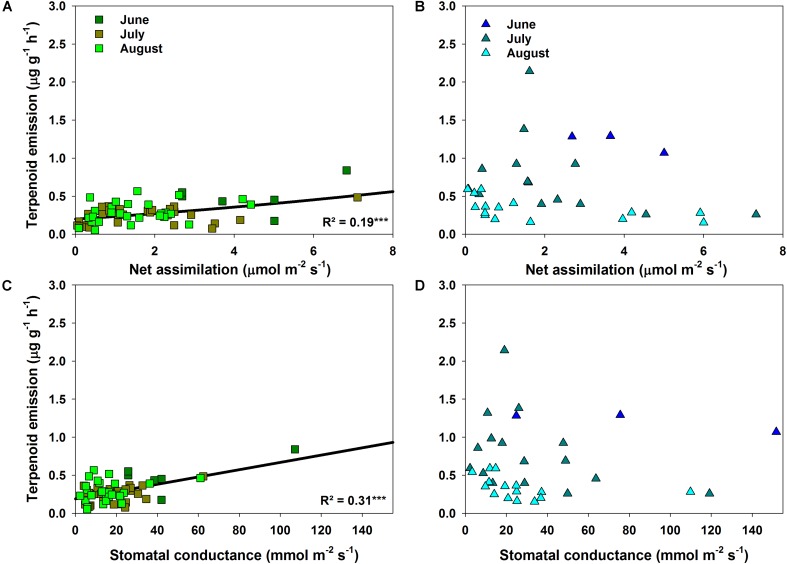
Relationship of measured terpenoid emission rates for *Q. suber* **(A,C)** and standardized terpenoid emission rates for *C. ladanifer* **(B,D)** to net assimilation rate and stomatal conductance. Significant trends (linear and exponential regressions) are illustrated by solid lines at a significance levels of ^∗^*p* < 0.05, ^∗∗^*p* < 0.01, ^∗∗∗^*p* < 0.001.

The correlation between terpenoid emissions and environmental factors during drought differed clearly between species. *Q. suber* revealed only a slight, non-significant decrease in emissions with declining Ψ_PD_ (**Figure 4A**). Terpenoid emissions of *C. ladanifer*, on the other hand, were significantly (*p* < 0.001) negatively correlated with decreasing Ψ_PD_ (**Figure 4B**). Similarly, increasing air temperatures also exerted a significant positive influence on terpenoid emissions (**Figure 4D**). Especially in July, when the highest air temperatures occurred, the pronounced exponential increase of emissions at higher air temperatures was highly significant (*R*^2^ = 0.68, *p* < 0.001). Hence, terpenoid emissions of *C. ladanifer* were temperature-standardized for further analysis to account for the variability caused by diurnal air temperature variations. Similar to Ψ_PD_, the relationship of emissions and air temperature was weak in *Q. suber* (**Figure 4C**). At most, a slight, non-significant tendency of decreasing emissions with increasing air temperature was evident in June; whereas in July and August no correlation was found. However, there was a weak, yet significant correlation of assimilation rates and stomatal conductance with terpenoid emissions of *Q. suber* (**Figures 5A,C**). Hence, more terpenoids were released at higher carbon assimilation and stomatal conductance. Due to the high degree of correlation of stomatal conductance and assimilation rates (*R*^2^ = 0.76), the influence of each individual factor on terpenoid emissions is difficult to evaluate. In addition, results have to be interpreted with care as the *R*^2^ of regressions were low (**Figures 5A,C**). *C. ladanifer* showed decreasing carbon assimilation and stomatal conductance in the sampling period, yet no correlation with standardized or measured terpenoid emissions was found (**Figures 5B,D** and Supplementary Figures 1A,B).

### Standard Emission Factors of Single Terpenoid Compounds

**FIGURE 6:**
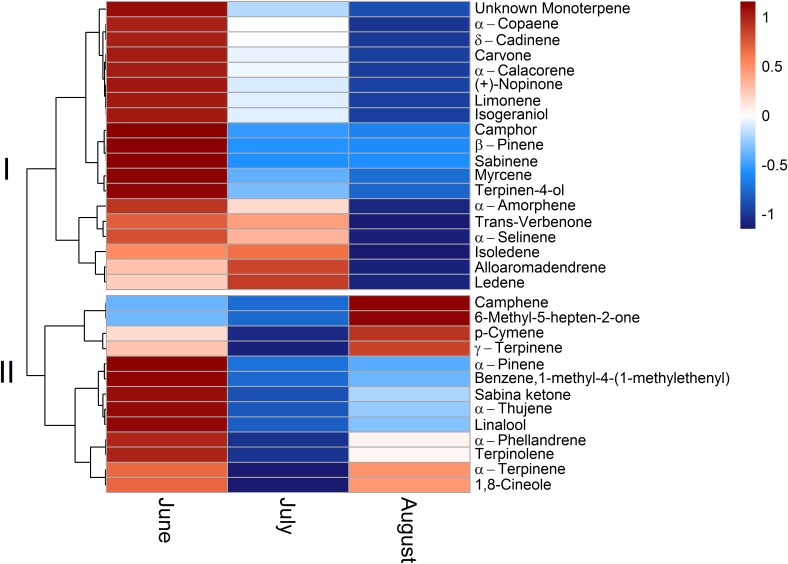
Relative change of single terpenoid compound emissions over time for *Q. suber* illustrated as clustered heat maps. Emissions are standardized to a temperature of 30°C (Guenther et al., 1993). The color code indicates the relative changes of emissions over time. Red colors indicate increasing emission rates on a scale from 0 to 1; where a color code of 0 corresponds to unaffected emission rates and a color code of 1 corresponds to strongly increased emission rates. Blue colors indicate decreasing emission rates on a scale from 0 to –1, where a color code of 0 corresponds to unaffected emission rates and a color code of –1 corresponds to strongly decreased emission rates.

**FIGURE 7:**
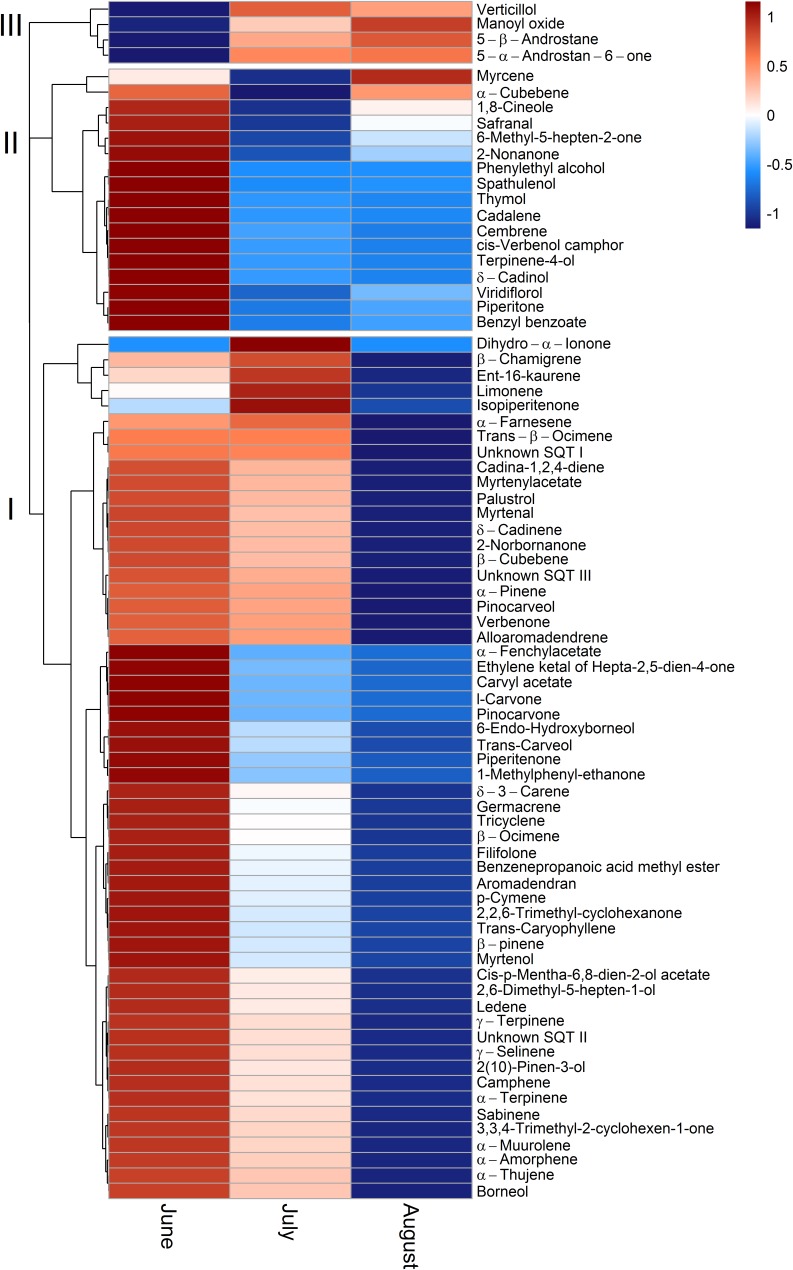
Relative change of single terpenoid compound emissions over time for *C. ladanifer* illustrated as clustered heat maps. Emissions are standardized to a temperature of 30°C (Guenther et al., 1993). The color code indicates the relative changes of emissions over time. Red colors indicate increasing emission rates on a scale from 0 to 1; where a color code of 0 corresponds to unaffected emission rates and a color code of 1 corresponds to strongly increased emission rates. Blue colors indicate decreasing emission rates on a scale from 0 to –1, where a color code of 0 corresponds to unaffected emission rates and a color code of –1 corresponds to strongly decreased emission rates.

**FIGURE 8:**
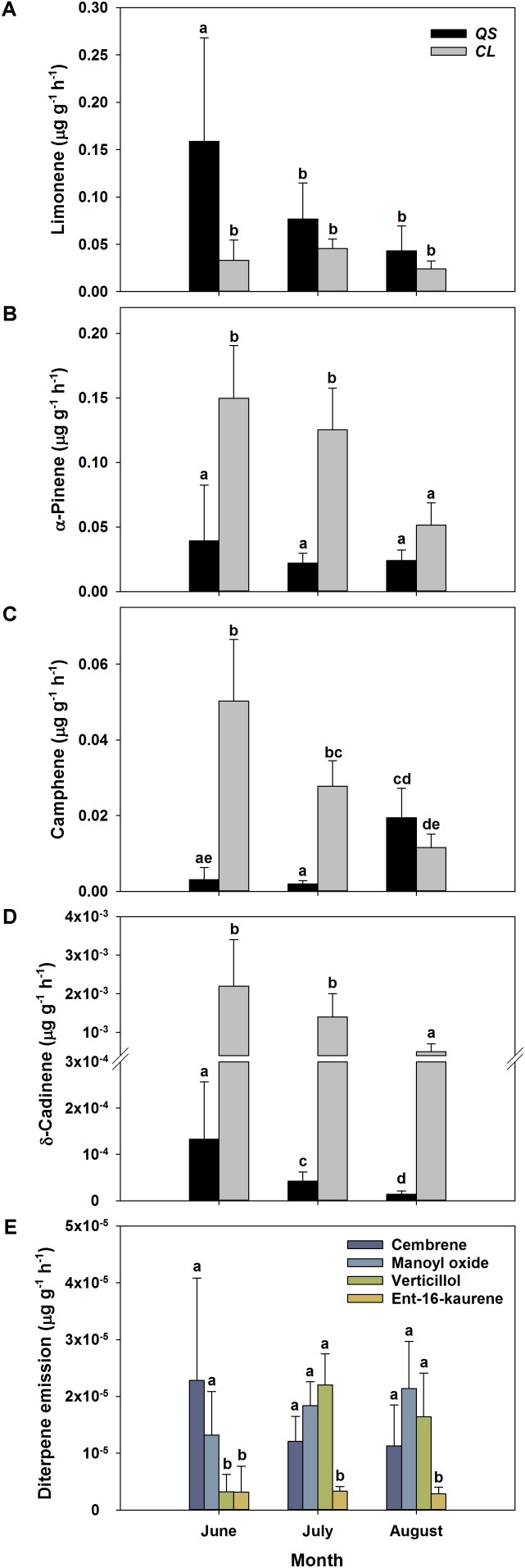
Absolute change of standard emissions factors (30°C, Guenther et al., 1993) of selected terpenoid compounds over time for *Q. suber* **(A–D)** (*n* = 18 for June, *n* = 32 for July and August) and *C. ladanifer* **(A–E)** (*n* = 9 for June, *n* = 16 for July and August) with a confidence interval of 95%. Selected compounds are the monoterpenes limonene **(A)**, α-pinene **(B)**, camphene **(C)**, the sesquiterpene alloaromadendrene **(D)** and the diterpenes cembrene, manoyl oxide, verticillol and ent-16-kaurene **(E)**. Compounds were selected according to their quantitative importance and development of emissions over time. Letters indicate statistical differences (RMANOVA) of selected terpenoid emissions between sampling periods and species at a significance level of *p* < 0.05.

To allow for a better comparability of emission rates with progressing drought, standard emission factors at an air temperature of 30°C were calculated. It must be denoted that no significant correlation of air temperature and terpenoid emission rates was found for total terpenoid emissions of *Q. suber*. Nevertheless, emissions were standardized to 30°C to allow for a comparison of standard emission factors of both species and literature. Due to the low temperature dependence, the standardization procedure did not alter the pattern of emission rates for MT and MTO emissions, as only SQT emissions of *Q. suber* did express a temperature dependence. For a comparison, calculations with non-standardized emissions of *Q*. *suber* are also shown in the appendix (Supplementary Figure 2). To identify common temporal patterns within the diversity of emitted terpenoids, heat maps clustering compounds with similar emission patterns, were created, showing the relative change in standard emission factors over time (**Figures 6**, **7**). Overall, increasing (red, 0 – 1), unaffected (white, 0), and decreasing (blue, 0 – -1) emission patterns of single terpenoid compounds were found, which could be assigned to two main clusters for *Q. suber* (**Figure 6**) and three main cluster for *C. ladanifer* (**Figure 7**). The biggest cluster (I) in both species was characterized by different terpenoids whose emissions decreased with progressive drought (**Figures 6**, **7**), and dominated the total terpenoid emissions, as already evident from **Figures 3**, **4**. Most of the emitted SQTs, such as δ-cadinene (**Figure 8D**) could be allocated to this cluster, but also MT such as β-pinene or sabinene (see also **Tables 1A,B**). Nevertheless, there were also a few compounds in this cluster with emission peaks in July, such as limonene or the DT ent-16-kaurene for *C. ladanifer* or the SQTs alloaromadendrene and ledene for *Q. suber*. However, those changes were non-significant (*p* > 0.05), as evident from **Figures 8A,E** for limonene and ent-16-kaurene emissions of *C. ladanifer*. The second cluster II was characterized by more irregular patterns of standard emissions factors (**Figures 6**, **7**). For *Q. suber*, cluster II contained compounds with lowest standard emission factors in July and increases somewhat thereafter, best illustrated by the MTs α-pinene, camphene and γ-terpinene (**Figures 6**, **8B,C** and **Table 1A**). While the changes of α-pinene were minor and non-significant (*p* > 0.05), the increase of camphene in August was highly significant (*p* < 0.001). Although, α-pinene and camphene did not show the same pattern in *C. ladanifer*, cluster II also contained compounds with lowest standard emission factor in July, such as myrcene or 1,8-cineole (**Figure 7**). However, there was a small, third cluster containing larger compounds, such as the DTs manoyl oxide and verticillol, whose standard emission factors increased progressively over time (**Figure 8E** and **Table 1B**). While this increase was not significant for manoyl oxide (*p* > 0.05), it was highly significant (*p* < 0.001) for verticillol from June to July. The standard emission factor of cembrene on the other hand decreased non-significantly from June to July (**Figure 8E**). Because heat maps only illustrate relative changes in emissions, the standard emission factors as well as the empirical temperature coefficients (β) of the most important terpenoid compounds are compiled in **Tables 1A,B** for each species. The only compound group which revealed a positive correlation with air temperature for *Q. suber* were SQT emissions, as evident from positive β-values. β-values of MT and MTO emissions were mostly negative and should be interpreted with care. On the other hand, β values of all terpenoid compound groups of *C. ladanifer* were positive and increased in the following order: MTs < MTOs < DTs < SQTs (**Table 1B**). Interestingly, β values of SQTs increased strongly over time from 0.111 ± 0.029 in June to 0.201 ± 0.037 in August. Manoyl oxide and cembrene had the highest β value amongst the four emitted diterpenes. A table of all emitted compounds including standard emission factor, empirical temperature coefficient (β), RI values and match factors of compound identification is given in Supplementary Tables 1, 2 of the Supplementary Material.

**Table 1:** Standard emission factor *E*_s_ (μg C g^-1^ h^-1^) with 95% confidence interval and empirical temperature coefficient β (°C^-1^) with 95% confidence interval for *Quercus suber* **(A)** and *Cistus ladanifer* **(B)**.

(A)					

Compound	*E*_s_	β	*E*_s_	β	*E*_s_	β
		
*Quercus suber*	June (*n* = 18)		July (*n* = 32)		August (*n* = 32)	
Total terpenoids	0.543 ± 0.208	-0.062 ± 0.038	0.224 ± 0.043	-0.021 ± 0.015	0.245 ± 0.047	-0.033 ± 0.023
Monoterpenes	0.456 ± 0.202	-0.068 ± 0.043	0.186 ± 0.039	-0.038 ± 0.016	0.216 ± 0.042	-0.036 ± 0.023
Oxy. Monoterpenes	0.053 ± 0.032	-0.047 ± 0.058	0.026 ± 0.009	0.035 ± 0.027	0.025 ± 0.007	-0.007 ± 0.031
Sesquiterpenes	5.32 × 10^-4^ ± 4.29 × 10^-4^	0.139 ± 0.077	2.56 × 10^-4^± 1.17 × 10^-4^	0.168 ± 0.036	3.53 × 10^-5^ ± 2.76 × 10^-5^	0.164 ± 0.091
Limonene	0.159 ± 0.109	-0.005 ± 0.067	0.077 ± 0.038	-0.059 ± 0.038	0.043 ± 0.026	-0.092 ± 0.071
α-Pinene	0.039 ± 0.043	-0.149 ± 0.107	0.022 ± 0.008	-0.084 ± 0.027	0.024 ± 0.008	-0.001 ± 0.040
β-Pinene	0.036 ± 0.033	-0.131 ± 0.091	0.012 ± 0.005	-0.068 ± 0.032	0.012 ± 0.004	-0.048 ± 0.043
γ-Terpinene	0.017 ± 0.014	-0.121 ± 0.077	0.007 ± 0.003	-0.030 ± 0.030	0.025 ± 0.008	-0.043 ± 0.038
Sabinene	0.031 ± 0.033	-0.151 ± 0.103	0.005 ± 0.003	-0.053 ± 0.041	0.005 ± 0.002	0.003 ± 0.053

**(B)**					

***Cistus ladanifer***	**June (*n* = 9)**		**July (*n* = 16)**		**August (*n* = 16)**	

Total terpenoids	1.027 ± 0.345	0.064 ± 0.033	0.590 ± 0.119	0.099 ± 0.017	0.298 ± 0.109	0.097 ± 0.043
Monoterpenes	0.630 ± 0.270	0.053 ± 0.042	0.428 ± 0.098	0.089 ± 0.019	0.223 ± 0.084	0.087 ± 0.044
Oxy. Monoterpenes	0.181 ± 0.051	0.038 ± 0.027	0.092 ± 0.015	0.092 ± 0.014	0.057 ± 0.021	0.111 ± 0.042
Sesquiterpenes	0.200 ± 0.060	0.111 ± 0.029	0.063 ± 0.015	0.149 ± 0.020	0.014 ± 0.005	0.201 ± 0.037
Diterpenes	4.98 × 10^-5^ ± 3.34 × 10^-5^	0.116 ± 0.066	6.04 × 10^-5^ ± 1.09 × 10^-5^	0.084 ± 0.015	5.98 × 10^-5^ ± 2.29 × 10^-5^	0.135 ± 0.045
γ-Terpinene	0.208 ± 0.129	0.053 ± 0.060	0.135 ± 0.032	0.096 ± 0.020	0.068 ± 0.028	0.121 ± 0.047
α-Pinene	0.150 ± 0.041	0.043 ± 0.027	0.125 ± 0.032	0.074 ± 0.022	0.051 ± 0.017	0.057 ± 0.040
Limonene	0.033 ± 0.021	0.082 ± 0.061	0.046 ± 0.010	0.086 ± 0.019	0.024 ± 0.008	0.074 ± 0.041
2,6-dimethyl-5-hepten-1-ol	0.049 ± 0.022	0.032 ± 0.043	0.032 ± 0.009	0.059 ± 0.024	0.018 ± 0.007	0.125 ± 0.048
α-Terpinene	0.047 ± 0.044	0.039 ± 0.090	0.029 ± 0.008	0.093 ± 0.022	0.015 ± 0.004	0.140 ± 0.034
Manoyl oxide	1.32 × 10^-5^ ± 7.64 × 10^-6^	0.122 ± 0.056	1.83 × 10^-5^ ± 4.25 × 10^-6^	0.108 ± 0.020	2.14 × 10^-5^ ± 8.25 × 10^-6^	0.139 ± 0.045
Cembrene	2.28 × 10^-5^ ± 1.80 × 10^-5^	0.124 ± 0.076	1.21 × 10^-5^ ± 4.40 × 10^-6^	0.103 ± 0.031	1.13 × 10^-5^ ± 7.17 × 10^-6^	0.131 ± 0.075
Verticillol	3.23 × 10^-6^ ± 3.03 × 10^-6^	0.079 ± 0.093	2.20 × 10^-5^ ± 5.49 × 10^-6^	0.063 ± 0.021	1.64 × 10^-5^ ± 7.69 × 10^-6^	0.130 ± 0.055
Ent-16-kaurene	3.13 × 10^-6^ ± 4.60 × 10^-6^	0.089 ± 0.142	3.31 × 10^-6^ ± 8.33 × 10^-7^	0.066 ± 0.021	2.84 × 10^-6^ ± 1.16 × 10^-6^	0.119 ± 0.048


*E_*s*_ and β were calculated according to Guenther et al. (1993) (see equation 2), where E_*s*_ is the y-intercept and β the slope of the regression. For Q. suber the different terpenoid compound groups and the five terpenoid compounds with the highest emission rates are displayed, for C. ladanifer single diterpene compounds are additionally displayed*.

## Discussion

Our results show that the decline in terpenoid emissions from co-occurring *Q. suber* and *C. ladanifer* during summer was significantly correlated with increasing drought stress. This correlation was clearly species-specific, which showed differences in terpenoid emission rates, emitted compounds and reaction to environmental conditions (water stress and diurnal temperature changes). Standard emission factors revealed interesting patterns of decreasing, unaffected and increasing emission rates of individual terpenoid compounds with increasing summer drought.

### Terpenoid Emissions in Relation to Drought Adaptation Strategies

*Cistus ladanifer* was not only characterized by higher terpenoid emission rates than *Q. suber*, but, in particular, by a high diversity of over 75 different compounds. Overall, the emission rates observed in our study were in the lower range of emissions reported earlier for *Cistus* spp. (3 – 21 μg g^-1^ h^-1^, Pio et al., 1993; Ormeno et al., 2007; Lluisà et al., 2010). Compared to *Q. suber*, *C. ladanifer* showed a stronger responsiveness to changing environmental conditions. This pattern is in agreement with the characteristic adaptation strategy of this species to withstand summer drought. Low thresholds for xylem cavitation and hydraulic failure (Quero et al., 2011) allow a high physiological activity, even when water resources start to decline (Núñez-Olivera et al., 1996; Ramírez et al., 2012). As terpenoid production is dependent on plant metabolism (e.g., Kesselmeier and Staudt, 1999), it is likely that *C. ladanifer* was able to synthesize a substantial amount of terpenoids in June, when assimilation rates were still quite high (Ramírez et al., 2012). Most probably, these compounds were either emitted directly or maintained in the storage pools of terpenoids present in the leaves of this species (Alías et al., 2012), as also demonstrated for *C. albidus* and *C. monspeliensis* under drought conditions (Lluisà and Peñuelas, 1998; Lluisà et al., 2010). Lluisà and Peñuelas (1998) suggested that plants under mild to moderate drought stress accumulate carbon which is then often allocated to defense compounds such as terpenoids, when growth is restricted by water limitation. The emission of stored terpenoids is mostly temperature dependent (Lluisà and Peñuelas, 1998; Staudt et al., 2017), because the volatility of these compounds increases at higher air temperatures (Lerdau et al., 1997; Peñuelas and Lluisà, 2001), which is supported here by the significant correlation with diurnal temperature variations (**Figure 4D**). Nevertheless, with prolonged drought conditions terpenoid fluxes declined, which was clearly visible in the seasonal patterns of standard emission factors and in line with declining Ψ_PD_ and carbon assimilation, indicating an increase of shrub and tree drought stress over time (Grassi and Magnani, 2005). In the absence of severe drought stress seasonal terpenoid emissions have been shown to increase from spring to summer for *Cistus* spp. (Lluisà and Peñuelas, 2000; Rivoal et al., 2010) and *Q. suber* (Staudt et al., 2004; Pio et al., 2005), only declining in autumn, probably caused by lower air temperatures and leaf senescence. Hence, it is likely that severe drought stress was the determining factor for the observed reduction in terpenoid emissions. Although no direct link between carbon assimilation and terpenoid emissions was found in *C. ladanifer*, diminished substrate availability for terpenoid biosynthesis probably contributed to the overall decline of emissions. With increasing drought stress, assimilation rates were stronger reduced compared to those of *Q. suber* as a result of the opportunistic, water-spending strategy of *C. ladanifer.* Compound concentrations in storage pools possibly got depleted by high air temperatures and cumulative drought stress over time and were not refilled to the same level (Lluisà et al., 2006; Staudt et al., 2017), as evident from SQT emissions which are known to be stored in leaves of *Cistus* spp. (Lluisà and Peñuelas, 2000; Ormeno et al., 2007). There is also substantial evidence, that terpenoid storing species may release terpenoids from *de novo* biosynthesis dependent on substrate availability from photosynthesis (Lluisà et al., 2010; Staudt et al., 2017), which decreased strongly in *C. ladanifer* over time. Since the diurnal temperature dependence, as well as the decrease of MT emissions with drought was lower compared to SQT, it seems likely that a substantial amount of MTs originated from *de novo* biosynthesis, as recently suggested by Yáñez-Serrano et al. (2018). However, these effects cannot be disentangled from our field measurements, where emissions might have been influenced by other co-varying factors, such as PPFD or biotic interactions. Nevertheless, studies of terpenoid emissions in a natural environment are rare and illustrate actual emission patterns more closely than controlled environment experiments.

*Quercus suber* pursues a different strategy to cope with water scarcity compared to *C. ladanifer*. The access to deep water resources with a tap root system and stomatal control over transpiration (David et al., 2007) allow this species to maintain relatively high water potentials and to minimize transpiration losses, probably to avoid hydraulic failure. However, this strategy leads to reduced carbon assimilation rates and the typical midday depression of gas exchange (e.g., Chaves, 1991) and affects the metabolism of plants. As *Q. suber*, in contrast to *C. ladanifer*, does not possess specialized storage organs for terpenoids in the leaves, emissions are assumed to be almost completely dependent on *de novo* biosynthesis (Loreto et al., 1996). Hence, lower emission rates in periods of water scarcity are most likely a direct consequence of this drought avoiding strategy and the lack of specialized terpenoid storage pools. However, in contrast to our assumptions, terpenoid emissions of *Q. suber* were not strongly dependent on Ψ_PD_ and air temperatures within the sampling period, as observed in closely related species such as *Q. ilex* (e.g., Lavoir et al., 2009). Thus, most likely, *Q. suber* did respond to the early onset of drought in June, as Ψ_PD_, Ψ_MD_ and sap flux density indicate that trees were already trying to avoid substantial water losses, possibly to prevent hydraulic failure (Pinto et al., 2012; Kurz-Besson et al., 2014). In agreement with this assumption, net assimilation rates of *Q. suber* of 6.12 ± 0.42 μmol m^-2^ s^-1^ were already reduced in June, compared to typical spring values of 13 – 14 μmol m^-2^ s^-1^ (Tenhunen et al., 1985; Kurz-Besson et al., 2014), but more stable than assimilation rates of the water spending shrub *C. ladanifer*. The tendency of declining emissions at higher air temperatures in June, especially for MTs and MTOs, can be regarded as an interaction of drought and heat stress, which can lead to an inhibition of enzymes involved in *de novo* emissions of terpenoids (Loreto and Schnitzler, 2010). Hence, at higher air temperatures, primary substrate availability and terpenoid synthesis are reduced in non-storing species (Lavoir et al., 2009; Grote et al., 2010). Under severe drought, this response to air temperature is offset in non-storing plants (Brilli et al., 2007; Fortunati et al., 2008), which explains the pattern observed in July and August. A further indication that drought conditions were nevertheless an important determinant of emissions is given by the weak relationship of terpenoid emissions and carbon assimilation, which are usually highly correlated in non-storing species (Kesselmeier and Staudt, 1999; Niinemets et al., 2014). This finding further points toward a limitation in the terpenoid synthesis pathway. Although SQT emissions were low and declined strongly in *Q. suber*, they expressed a high temperature dependence, comparable to the SQTs emitted by *C. ladanifer*. Hence, it seems possible that SQTs were not only emitted from *de novo* biosynthesis, but also from small, probably temporary, storage pools in leaves of *Q. suber* (Pio et al., 2005). Noteworthy, the terpenoid emissions of *Q. suber* in our study were low compared to previously published results (10 – 43 μg g^-1^ h^-1^, Staudt et al., 2004; Pio et al., 2005; Staudt et al., 2008; Bracho-Nunez et al., 2013). As stated above, such low emission rates were most likely a consequence of the predominant environmental conditions during spring and summer 2017, which were characterized by very low precipitation compared to the long-term average, in combination with high air temperatures during the terpenoid sampling dates. Hence, to further unravel the effect of severe drought on terpenoid emissions of *Q. suber* and *C. ladanifer*, more studies characterizing emission patterns in pre-drought and recovery periods are required. Next to the different environmental conditions present in our experiments compared to other studies on terpenoid emissions of *Q. suber* (Staudt et al., 2004, 2008; Pio et al., 2005; Bracho-Nunez et al., 2013) and *Cistus* spp. (Pio et al., 1993; Ormeno et al., 2007; Lluisà et al., 2010), it must be denoted that emission rates may be underestimated for those terpenoids which show fast reaction with ozone (Vickers et al., 2009), because we did not use ozone scrubbers during terpenoid sampling in the field. Apart from varying environmental conditions, other differences in methodology, such as sampling flow rate, enclosure volume, terpenoid storage and analysis were minor and thus, should have had a negligible effect on terpenoid emission rates.

### Significance of Emitted Compounds for Stress Adaptation

In contrast to the decline in total terpenoid emission rates with enhanced drought stress over the season, emission of some individual terpenoid species was unaffected or even increased in both species, as evident from standard emission factors. Among these compounds were the MTs α-pinene, camphene and γ-terpinene (**Figure 6**). Standard emissions of α-pinene, a main compound emitted by both investigated species, were essentially unaffected in *Q. suber* and partly also in *C. ladanifer*. Camphene and γ-terpinene were emitted even at higher rates in *Q. suber* in August (**Figure 8C** and **Table 1A**). As emission of terpenoids represents a carbon loss (e.g., Vickers et al., 2009), this investment is likely to have a beneficial effect for the plants (Possell and Loreto, 2013). In contrast, emissions of other terpenoids such as limonene, the dominant MT of *Q. suber* in Portugal (Loreto et al., 2009), were decreasing continuously, indicating their minor role in stress adaptation, as also suggested by Lluisà et al. (2005) for limonene. It is well understood that prolonged drought stress in combination with high air temperatures and light intensities can increase the abundance of reactive oxygen species in leaves (e.g., Miller et al., 2008), which potentially damages the photosynthetic apparatus of plants (Peñuelas et al., 2005; Velikova and Loreto, 2005). Particularly for the long-lived leaves of *Q. suber*, any damage would be costly for trees (Loreto et al., 2014); thus these evergreen Mediterranean trees have developed multiple strategies to protect these organs (Werner et al., 2002). Hence, when preventing hydraulic failure by stomatal closure, those terpenoids showing increased emission rates might play a particular role to avoid permanent damage to the photosynthetic apparatus under stressful conditions (Vickers et al., 2009; Loreto et al., 2014), e.g., by maintaining the stability of thylakoid membranes (Velikova et al., 2011, 2012). Specific terpenoids therefore might provide effective protection during summer drought (Delfine et al., 2000; Loreto et al., 2004; Copolovici et al., 2005; Possell and Loreto, 2013). MT emission seems to be a general feature of Mediterranean plants, which supports the assumption that they provide a strategy to withstand drought, heat and light stress.

Leaves of *C. ladanifer* on the other hand, are reported to remain photo-protected and potentially active during the early onset of summer drought, to take immediate advantage of favorable conditions such as short summer rains (Núñez-Olivera et al., 1996; Ramírez et al., 2012). To this end, higher carotenoid content in leaves, exudation of flavonoids and DTs and the incorporation of phenolic substances into the xylem have been reported as effective protection measures against oxidative stress (Núñez-Olivera et al., 1996; De Micco and Aronne, 2007; Valares Masa et al., 2016). Hence, the diverse blend of terpenoids detected in the emissions of this species likely also contributes to the high drought tolerance of *C. ladanifer.* Whereas the role of MTs in stress response is quite well established, the role of SQTs is less well understood (Vickers et al., 2009). Decreasing release of SQTs, such as δ-cadinene with progressing drought, suggests that the main function of SQT is not solely abiotic stress adaptation. Several SQT compounds have been identified in the allelopathic oil of *C. ladanifer* (Gomes et al., 2005) indicating an important role in biotic interactions (Verdeguer et al., 2012). Even more intriguing is the role of DTs in terpenoid emissions of *C. ladanifer*. Although only small emission rates occurred, DTs were so far rather considered as semi-volatile or non-volatile (Dudareva et al., 2006; Niinemets, 2010; Loreto et al., 2014) and indeed there are very few studies which report volatile diterpenoid emissions (Otsuka et al., 2004; Von Schwartzenberg et al., 2004; Matsunga et al., 2012). However, recently Yáñez-Serrano et al. (2018) detected significant emissions of the DT ent-16-kaurene not only from *C. ladanifer*, but also from *Halimium halimifolium*, a related Mediterranean shrub species. Moreover, the DTs manoyl oxide and ent-16-kaurene are known as major components in essential oils of other related species such as *C. monspeliensis* and *C. creticus* (Demetzos et al., 1997; Angelopoulou et al., 2002), which could indicate that DT emissions may occur from more Mediterranean species than previously thought. The ecological role of DT emissions is yet to be determined, but there is evidence that they are involved in photoprotective mechanisms during stressful periods (Munné-Bosch and Alegre, 2000; Munné-Bosch et al., 2001) and possess allelopathic and antimicrobial properties (Demetzos et al., 1997; Alías et al., 2012). Increasing and unaffected emissions of verticillol, manoyl oxide and ent-16-kaurene under progressing drought might indeed indicate an important role of DTs in abiotic stress adaptation. Hence, the compound-rich blend of biochemicals identified in *C. ladanifer* might provides a competitive advantage for this species to withstand stressful periods (De Micco and Aronne, 2007; Valares Masa et al., 2016). Next to the scarce knowledge about DT emissions, the influence of these long-chained terpenoids on atmospheric chemistry is yet to be determined (Otsuka et al., 2004; Yáñez-Serrano et al., 2018). While the reaction rate constant toward ozone and hydroxyl radicals is low for ent-16-kaurene (1.2 × 10^-17^cmł molec^-1^ s^-1^ and 72.5 × 10^-12^cmł molec^-1^ s^-1^) and manoyl oxide (1.8 × 10^-18^ cmł molec^-1^ s^-1^ and 56.7 × 10^-12^cmł molec^-1^ s^-1^) (EPI Suite, Environmental Protection Agency, United States), verticillol and cembrene are assumed to react faster. The reaction rate constant toward ozone is approximately twofold higher for verticillol (86.0 × 10^-17^cmł molec^-1^ s^-1^) and fourfold higher for cembrene (186.0 × 10^-17^cmł molec^-1^ s^-1^) compared to β-caryophyllene (44.2 × 10^-17^cmł molec^-1^ s^-1^) or α-pinene (43.0 × 10^-17^cmł molec^-1^ s^-1^). In comparison to isoprene (105.1 × 10^-12^cmł molec^-1^ s^-1^), verticillol (201.6 × 10^-12^cmł molec^-1^ s^-1^) and cembrene 375.7 × 10^-12^cmł molec^-1^ s^-1^) are assumed to react approximately two and four times faster toward hydroxyl radicals (EPI Suite, Environmental Protection Agency, United States). Thus, given the large impact of other terpenoids in ozone production and aerosol formation (Holopainen and Gershenzon, 2010), DT emissions might amplify the impact of terpenoids on atmospheric chemistry.

## Conclusion

Our results suggest a species- and terpenoid-specific behavior of severe drought and terpenoid emissions. *Q. suber* and *C. ladanifer* differed strongly in relation to the diversity of emissions and reactions to assimilation rates, water potentials and diurnal air temperature variations. While overall terpenoid emissions strongly decreased over time, unaffected or increasing emissions of some terpenoid compounds illustrate the importance of terpenoids in drought adaptation.

## Author Contributions

SH conducted the field work, statistical analysis, and wrote the manuscript. JK planned the experiment, performed the TD-GC-MS analysis, and processed the data. RL-d-V and MC helped in planning the experiment, conducted the field work, and assisted the interpretation of the data. MD helped in planning the experiment and interpretation of the data. CW planned the experiment, assisted the field work and the interpretation of the data. All authors critically discussed and reviewed the manuscript.

## Conflict of Interest Statement

The authors declare that the research was conducted in the absence of any commercial or financial relationships that could be construed as a potential conflict of interest.

## Footnotes

1 www.ipma.pt

2 www.ipma.pt
