# Supplementary material for: Terpenoid Emissions of Two Mediterranean Woody Species in Response to Drought Stress
Source: Front Plant Sci. 2018 Jul 23;9:1071. doi: 10.3389/fpls.2018.01071 (PMC6064746; doi:10.3389/fpls.2018.01071)
Supplement: Supplementary file 1 [file Data_Sheet_1.pdf]

Supplementary Material

Terpenoid emissions of two Mediterranean woody species in response to drought stress

Simon Haberstroh[1,2*], Jürgen Kreuzwieser[1], Raquel Lobo-do-Vale[2], Maria C. Caldeira[2], Maren Dubbert[1], Christiane Werner[1]

[1] Ecosystem Physiology, University Freiburg, Freiburg, Germany

[2] Centro de Estudos Florestais, Instituto Superior de Agronomia, Universidade de Lisboa, Lisboa, Portugal

*** Correspondence:**
Corresponding Author
simon.haberstroh@cep.uni-freiburg.de

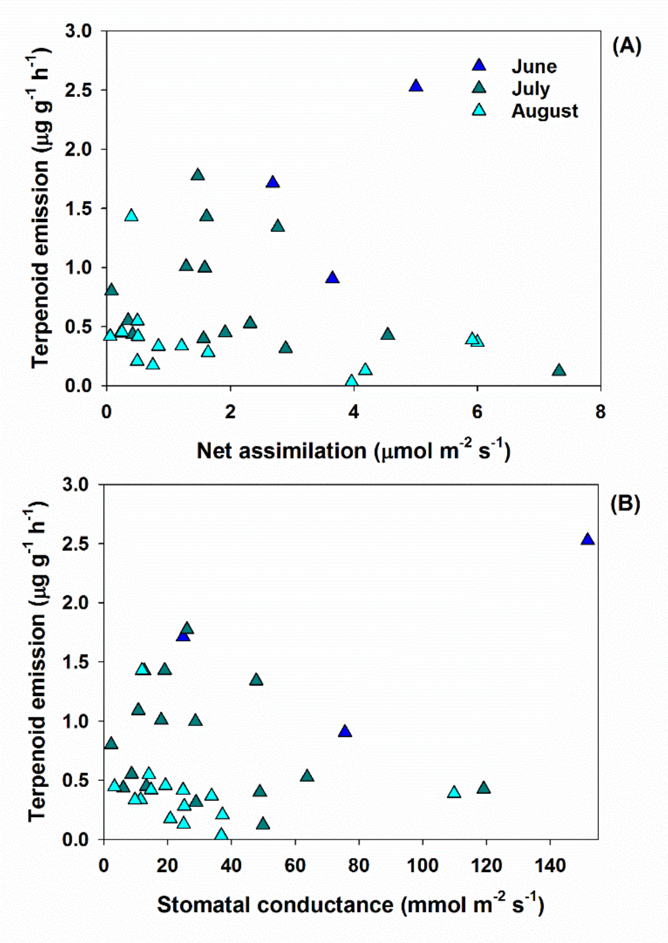

Supplementary Figure 1. Relationship of measured terpenoid emissions to net assimilation rate (A) and stomatal conductance (B) for *C. ladanifer*.

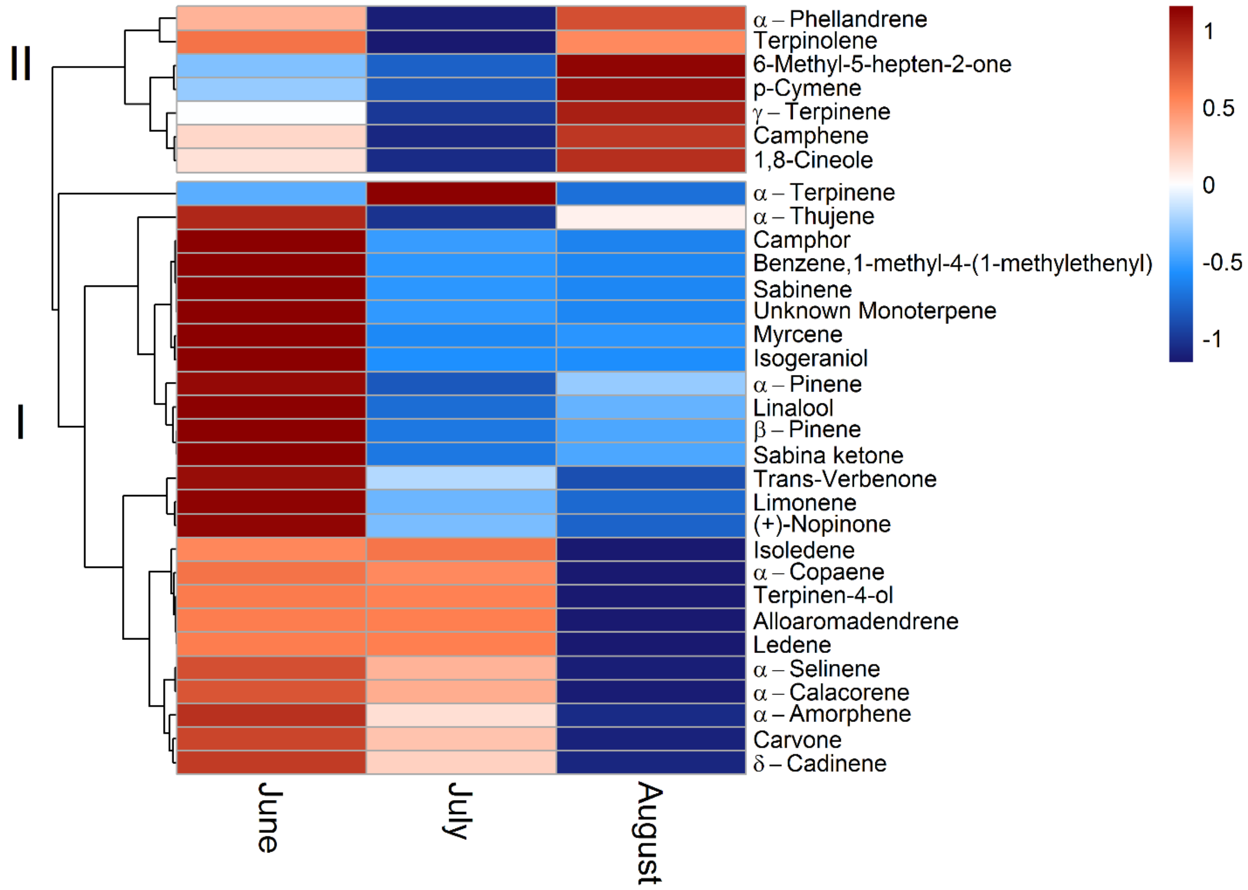

Supplementary Figure 2. Relative change of single terpenoid compound emissions over time for *Q. suber* illustrated as clustered heat maps. Emissions are measured emission rates. The colour code indicates the relative changes of emissions over time. Red colours indicate increasing emission rates on a scale from 0 to 1; where a colour code of 0 corresponds to unaffected emission rates and a colour code of 1 corresponds to strongly increased emission rates. Blue colours indicate decreasing emission rates on a scale from 0 to -1, where a colour code of 0 corresponds to unaffected emission rates and a colour code of -1 corresponds to strongly decreased emission rates.

Supplementary Table 1. Standard emission factor E_s (µg C g^{-1} h^{-1}) with 95 % confidence interval and empirical temperature coefficient β (°C^{-1}) with 95 % confidence interval, match factor (MF) and retention index (RI) of all emitted terpenoid compounds for *Q. suber* (n = 18 for June, n = 32 for July and August). E_s and β were calculated according to Guenther et al. (1993), where E_s is the y-intercept and β the slope of the regression.

Compound	Month	E_s	β	MF	RI
α-Thujene	June	$9.98 \times 10^{-3} \pm 9.21 \times 10^{-3}$	-0.125±0.090	97.5	931
	July	$2.96 \times 10^{-3} \pm 1.54 \times 10^{-3}$	-0.092±0.040		
	August	$4.25 \times 10^{-3} \pm 1.92 \times 10^{-3}$	-0.065±0.053		
α-Pinene	June	0.039±0.043	-0.149±0.107	76.2	938
	July	0.022±0.008	-0.084±0.027		
	August	0.024±0.008	-0.001±0.040		
Camphene	June	$3.11 \times 10^{-3} \pm 3.16 \times 10^{-3}$	-0.009±0.104	98.1	953
	July	$1.98 \times 10^{-3} \pm 8.50 \times 10^{-4}$	-0.074±0.033		
	August	$1.94 \times 10^{-2} \pm 7.77 \times 10^{-3}$	0.012±0.046		
β-Pinene	June	0.036±0.033	-0.131±0.058	97.3	979
	July	0.012±0.005	-0.068±0.032		
	August	0.012±0.004	-0.048±0.043		
Myrcene	June	$6.60 \times 10^{-3} \pm 3.91 \times 10^{-3}$	-0.060±0.058	97.9	991
	July	$2.37 \times 10^{-3} \pm 5.77 \times 10^{-4}$	-0.003±0.019		
	August	$1.90 \times 10^{-3} \pm 6.80 \times 10^{-4}$	-0.005±0.043		
Sabinene	June	0.031±0.033	-0.151±0.103	97.5	975
	July	0.005±0.003	-0.053±0.041		
	August	0.005±0.002	0.003±0.053		
6-Methyl-5-hepten-2-one	June	$1.95 \times 10^{-3} \pm 1.32 \times 10^{-3}$	0.085±0.066	90.1	987
	July	$1.54 \times 10^{-3} \pm 5.38 \times 10^{-4}$	0.047±0.027		
	August	$4.68 \times 10^{-3} \pm 2.84 \times 10^{-3}$	0.103±0.073		

**Compound**	**Month**	**E_s**	**β**	**MF**	**RI**
α-Phellandrene	June	$5.65 \times 10^{-3} \pm 3.19 \times 10^{-3}$	-0.068±0.058	97.1	1006
	July	$1.41 \times 10^{-3} \pm 7.38 \times 10^{-4}$	0.050±0.040		
	August	$2.98 \times 10^{-3} \pm 1.85 \times 10^{-3}$	-0.005±0.073		
α-Terpinene	June	$5.51 \times 10^{-4} \pm 3.67 \times 10^{-4}$	-0.057±0.065	97.6	1018
	July	$5.09 \times 10^{-4} \pm 2.00 \times 10^{-4}$	0.022±0.030		
	August	$5.46 \times 10^{-4} \pm 1.35 \times 10^{-4}$	0.022±0.029		
p-Cymene	June	$5.52 \times 10^{-3} \pm 2.45 \times 10^{-3}$	-0.020±0.043	98.7	1025
	July	$4.12 \times 10^{-3} \pm 1.39 \times 10^{-3}$	0.000±0.026		
	August	$6.62 \times 10^{-3} \pm 1.35 \times 10^{-3}$	-0.038±0.024		
Limonene	June	0.159±0.109	-0.005±0.067	92.8	1033
	July	0.077±0.038	-0.059±0.038		
	August	0.043±0.026	-0.092±0.071		
γ-Terpinene	June	0.017±0.014	-0.121±0.077	98.2	1061
	July	0.007±0.003	-0.030±0.030		
	August	0.025±0.008	-0.043±0.038		
Unknown Monoterpene	June	$9.21 \times 10^{-4} \pm 4.55 \times 10^{-4}$	-0.130±0.047	-	1060
	July	$1.65 \times 10^{-4} \pm 7.30 \times 10^{-5}$	-0.018±0.033		
	August	$6.56 \times 10^{-5} \pm 4.43 \times 10^{-5}$	-0.125±0.075		

Compound	Month	E_s	β	MF	RI
Benzene,1-methyl-4-(1-methylethenyl)	June	$8.99 \times 10^{-3} \pm 3.30 \times 10^{-3}$	-0.003±0.036	95.9	1090
	July	$5.25 \times 10^{-3} \pm 1.84 \times 10^{-3}$	0.026±0.027		
	August	$5.85 \times 10^{-3} \pm 1.38 \times 10^{-3}$	-0.061±0.027		
1,8-Cineole	June	$2.53 \times 10^{-3} \pm 1.48 \times 10^{-3}$	-0.048±0.057	85.0	1032
	July	$1.56 \times 10^{-3} \pm 5.40 \times 10^{-4}$	-0.014±0.027		
	August	$2.37 \times 10^{-3} \pm 1.19 \times 10^{-3}$	0.031±0.058		
Terpinolene	June	$7.61 \times 10^{-3} \pm 4.63 \times 10^{-3}$	-0.126±0.060	98.7	1086
	July	$2.84 \times 10^{-3} \pm 7.14 \times 10^{-4}$	-0.037±0.020		
	August	$4.75 \times 10^{-3} \pm 1.10 \times 10^{-3}$	-0.075±0.027		
Linalool	June	$1.49 \times 10^{-3} \pm 2.13 \times 10^{-3}$	-0.103±0.142	-	1090
	July	$6.68 \times 10^{-4} \pm 3.18 \times 10^{-4}$	0.057±0.037		
	August	$8.24 \times 10^{-4} \pm 5.57 \times 10^{-4}$	0.054±0.075		
Isogeraniol	June	$2.47 \times 10^{-3} \pm 3.56 \times 10^{-3}$	-0.048±0.141	75.6	1131
	July	$9.39 \times 10^{-4} \pm 7.26 \times 10^{-4}$	0.029±0.060		
	August	$4.33 \times 10^{-4} \pm 3.10 \times 10^{-4}$	0.035±0.085		
(+)-Nopinone	June	$2.34 \times 10^{-3} \pm 3.12 \times 10^{-3}$	0.008±0.132	93.2	1140
	July	$9.03 \times 10^{-4} \pm 6.68 \times 10^{-4}$	0.070±0.058		
	August	$4.40 \times 10^{-4} \pm 3.32 \times 10^{-4}$	-0.006±0.092		
Camphor	June	$4.18 \times 10^{-4} \pm 2.61 \times 10^{-4}$	-0.011±0.059	81.6	1148
	July	$9.22 \times 10^{-5} \pm 4.19 \times 10^{-5}$	-0.025±0.035		
	August	$8.28 \times 10^{-5} \pm 5.18 \times 10^{-5}$	-0.056±0.072		

Compound	Month	E_s	β	MF	RI
Sabina ketone	June	$2.33 \times 10^{-3} \pm 1.83 \times 10^{-3}$	0.013±0.077	96.3	1160
	July	$7.58 \times 10^{-4} \pm 4.07 \times 10^{-4}$	0.039±0.042		
	August	$1.11 \times 10^{-3} \pm 6.45 \times 10^{-4}$	0.075±0.067		
Terpinen-4-ol	June	$1.38 \times 10^{-2} \pm 1.58 \times 10^{-2}$	-0.041±0.110	96.4	1181
	July	$9.28 \times 10^{-3} \pm 5.27 \times 10^{-3}$	0.054±0.044		
	August	$8.24 \times 10^{-3} \pm 2.79 \times 10^{-3}$	-0.024±0.038		
Verbenone	June	$4.44 \times 10^{-4} \pm 3.58 \times 10^{-4}$	0.176±0.079	86.2	1229
	July	$4.04 \times 10^{-4} \pm 2.12 \times 10^{-4}$	0.003±0.043		
	August	$2.45 \times 10^{-4} \pm 1.36 \times 10^{-4}$	-0.033±0.071		
Carvone	June	$4.25 \times 10^{-4} \pm 2.84 \times 10^{-4}$	-0.077±0.062	76.4	1244
	July	$1.41 \times 10^{-4} \pm 4.59 \times 10^{-5}$	0.096±0.025		
	August	$5.59 \times 10^{-5} \pm 2.08 \times 10^{-5}$	-0.036±0.048		
α-Copaene	June	$2.43 \times 10^{-4} \pm 1.68 \times 10^{-4}$	0.083±0.064	80.0	1373
	July	$8.92 \times 10^{-5} \pm 4.31 \times 10^{-5}$	0.136±0.037		
	August	$3.23 \times 10^{-5} \pm 1.51 \times 10^{-5}$	0.098±0.054		
Isoledene	June	$1.49 \times 10^{-5} \pm 9.25 \times 10^{-6}$	0.133±0.058	76.0	1436
	July	$1.68 \times 10^{-5} \pm 9.45 \times 10^{-6}$	0.074±0.044		
	August	$2.54 \times 10^{-6} \pm 1.68 \times 10^{-6}$	0.089±0.067		

Compound	Month	E_s	β	MF	RI
Alloaromadendrene	June	$2.42 \times 10^{-5} \pm 2.14 \times 10^{-5}$	0.296±0.084	93.9	1456
	July	$5.32 \times 10^{-5} \pm 2.56 \times 10^{-5}$	0.122±0.037		
	August	$3.43 \times 10^{-6} \pm 1.99 \times 10^{-6}$	0.051±0.067		
Ledene	June	$3.59 \times 10^{-5} \pm 2.81 \times 10^{-5}$	0.224±0.071	87.8	1487
	July	$6.66 \times 10^{-5} \pm 4.13 \times 10^{-5}$	0.076±0.049		
	August	$1.02 \times 10^{-5} \pm 5.36 \times 10^{-6}$	0.026±0.064		
α-Selinene	June	$2.79 \times 10^{-5} \pm 2.63 \times 10^{-5}$	0.224±0.089	93.3	1491
	July	$1.33 \times 10^{-5} \pm 9.60 \times 10^{-6}$	0.180±0.056		
	August	$1.18 \times 10^{-6} \pm 5.06 \times 10^{-7}$	0.081±0.053		
α-Amorphene	June	$9.06 \times 10^{-5} \pm 8.00 \times 10^{-5}$	0.018±0.083	72.0	1508
	July	$1.84 \times 10^{-5} \pm 8.67 \times 10^{-6}$	0.122±0.037		
	August	$1.25 \times 10^{-6} \pm 2.22 \times 10^{-6}$	0.350±0.194		
δ-Cadinene	June	$1.33 \times 10^{-4} \pm 1.24 \times 10^{-4}$	0.052±0.088	84.5	1515
	July	$4.21 \times 10^{-5} \pm 2.00 \times 10^{-5}$	0.094±0.037		
	August	$1.37 \times 10^{-5} \pm 6.92 \times 10^{-6}$	0.005±0.059		
α-Calacorene	June	$6.92 \times 10^{-5} \pm 5.11 \times 10^{-5}$	0.029±0.068	80.8	1537
	July	$1.58 \times 10^{-5} \pm 7.35 \times 10^{-6}$	0.168±0.036		
	August	$4.27 \times 10^{-6} \pm 2.91 \times 10^{-6}$	0.123±0.085		

Supplementary Table 2. Standard emission factor E_s (µg C g^{-1} h^{-1}) with 95 % confidence interval and empirical temperature coefficient β (°C^{-1}) with 95 % confidence interval, match factor (MF) and retention index (RI) of all emitted terpenoid compounds for *C. ladanifer* (n = 9 for June, n = 16 for July and August). E_s and β were calculated according to Guenther et al. (1993), where E_s is the y-intercept and β the slope of the regression.

**Compound**	**Month**	**E_s**	**β**	**MF**	**RI**
Tricyclene	June	$1.23 \times 10^{-2} \pm 4.05 \times 10^{-3}$	0.047±0.032	98.8	926
	July	$5.57 \times 10^{-3} \pm 1.34 \times 10^{-3}$	0.087±0.020		
	August	$2.46 \times 10^{-3} \pm 9.06 \times 10^{-4}$	0.064±0.043		
α-Thujene	June	$7.27 \times 10^{-3} \pm 7.53 \times 10^{-3}$	0.055±0.100	96.6	932
	July	$4.50 \times 10^{-3} \pm 1.48 \times 10^{-3}$	0.129±0.028		
	August	$1.56 \times 10^{-3} \pm 8.38 \times 10^{-4}$	0.144±0.063		
α -Pinene	June	0.150±0.041	0.043±0.026	76.2	938
	July	0.125±0.032	0.074±0.022		
	August	0.051±0.017	0.057±0.040		

Compound	Month	E_s	β	MF	RI
Camphene	June	0.050±0.016	0.030±0.031	97.5	955
	July	0.028±0.007	0.093±0.021		
	August	0.012±0.004	0.074±0.036		
Sabinene	June	$1.51 \times 10^{-2} \pm 7.26 \times 10^{-3}$	0.070±0.046	97.5	975
	July	$7.76 \times 10^{-3} \pm 2.95 \times 10^{-3}$	0.103±0.032		
	August	$2.57 \times 10^{-3} \pm 1.52 \times 10^{-3}$	0.121±0.069		
β-Pinene	June	$3.37 \times 10^{-3} \pm 1.92 \times 10^{-3}$	0.084±0.055	97.3	979
	July	$2.67 \times 10^{-3} \pm 1.06 \times 10^{-3}$	0.103±0.033		
	August	$2.26 \times 10^{-3} \pm 9.66 \times 10^{-4}$	0.023±0.050		
6-Methyl-5-hepten-2-one	June	$4.11 \times 10^{-3} \pm 2.01 \times 10^{-3}$	0.109±0.047	90.1	987
	July	$7.03 \times 10^{-4} \pm 2.10 \times 10^{-4}$	0.064±0.025		
	August	$1.42 \times 10^{-3} \pm 1.05 \times 10^{-3}$	-0.039±0.087		
Myrcene	June	0.023±0.019	0.071±0.080	91.9	991
	July	0.009±0.003	0.124±0.023		
	August	0.045±0.017	0.014±0.043		
δ-3-Carene	June	$1.81 \times 10^{-2} \pm 1.26 \times 10^{-2}$	0.038±0.065	92.6	1007
	July	$8.43 \times 10^{-3} \pm 2.14 \times 10^{-3}$	0.111±0.021		
	August	$3.64 \times 10^{-3} \pm 1.94 \times 10^{-3}$	0.119±0.063		

Compound	Month	E_s	β	MF	RI
α-Terpinene	June	0.047±0.044	0.039±0.090	97.6	1018
	July	0.029±0.008	0.093±0.022		
	August	0.015±0.004	0.140±0.034		
p-Cymene	June	$1.69 \times 10^{-2} \pm 7.70 \times 10^{-3}$	0.020±0.044	82.6	1027
	July	$9.47 \times 10^{-3} \pm 1.73 \times 10^{-3}$	0.086±0.015		
	August	$6.01 \times 10^{-3} \pm 1.46 \times 10^{-3}$	0.112±0.029		
Limonene	June	0.033±0.021	0.082±0.061	92.8	1033
	July	0.045±0.010	0.086±0.019		
	August	0.024±0.008	0.074±0.041		
1,8-Cineole	June	$8.35 \times 10^{-3} \pm 4.35 \times 10^{-3}$	0.043±0.050	86.8	1034
	July	$4.63 \times 10^{-3} \pm 1.30 \times 10^{-3}$	0.076±0.024		
	August	$6.39 \times 10^{-3} \pm 4.71 \times 10^{-3}$	0.007±0.087		
2,2,6-Trimethyl-cyclohexanone	June	0.027±0.007	0.000±0.025	94.0	1037
	July	0.015±0.003	0.068±0.016		
	August	0.010±0.003	0.105±0.038		
Trans-β-Ocimene	June	$6.76 \times 10^{-3} \pm 2.43 \times 10^{-3}$	0.002±0.035	77.4	1048
	July	$6.75 \times 10^{-3} \pm 2.56 \times 10^{-3}$	0.051±0.032		
	August	$6.05 \times 10^{-3} \pm 3.59 \times 10^{-3}$	0.059±0.070		

Compound	Month	E_s	β	MF	RI
2,6-Dimethyl-5-hepten-1-ol	June	0.049±0.021	0.032±0.043	81.0	1054
	July	0.032±0.009	0.058±0.024		
	August	0.018±0.007	0.125±0.048		
β-Ocimene	June	$7.11 \times 10^{-3} \pm 3.49 \times 10^{-3}$	0.068±0.047	-	1055
	July	$3.39 \times 10^{-3} \pm 1.40 \times 10^{-3}$	0.121±0.035		
	August	$1.59 \times 10^{-3} \pm 7.62 \times 10^{-3}$	0.121±0.056		
γ-Terpinene	June	0.208±0.129	0.053±0.060	94.7	1061
	July	0.135±0.032	0.096±0.020		
	August	0.068±0.028	0.121±0.047		
Safranal	June	$1.57 \times 10^{-3} \pm 5.53 \times 10^{-4}$	-0.036±0.034	70.1	1072
	July	$5.43 \times 10^{-4} \pm 2.08 \times 10^{-4}$	0.074±0.032		
	August	$9.14 \times 10^{-4} \pm 2.25 \times 10^{-4}$	0.106±0.029		
3,3,4-Trimethyl-2-cyclohexen-1-one	June	$1.81 \times 10^{-3} \pm 8.72 \times 10^{-4}$	0.015±0.047	80.2	1080
	July	$7.09 \times 10^{-4} \pm 1.71 \times 10^{-4}$	0.074±0.020		
	August	$1.34 \times 10^{-4} \pm 4.30 \times 10^{-5}$	0.130±0.038		
2-Norbornanone	June	$2.25 \times 10^{-3} \pm 9.32 \times 10^{-4}$	-0.048±0.040	84.5	1085
	July	$8.22 \times 10^{-4} \pm 3.10 \times 10^{-4}$	0.081±0.032		
	August	$5.19 \times 10^{-5} \pm 3.44 \times 10^{-5}$	0.073±0.072		
2-Nonanone	June	$2.73 \times 10^{-3} \pm 1.23 \times 10^{-3}$	-0.157±0.046	90.3	1093
	July	$4.34 \times 10^{-4} \pm 3.10 \times 10^{-4}$	-0.029±0.060		
	August	$7.79 \times 10^{-4} \pm 2.86 \times 10^{-4}$	0.014±0.044		

Compound	Month	E_s	β	MF	RI
Filifolone	June	1.08 x 10^{-2}±7.19 x 10^{-3}	0.030±0.064	92.9	1102
	July	5.27 x 10^{-3}±1.25 x 10^{-3}	0.103±0.020		
	August	2.78 x 10^{-3}±8.95 x 10^{-3}	0.151±0.038		
Ethylene ketal of Hepta-2,5-dien-4-one	June	1.07 x 10^{-2}±4.26 x 10^{-3}	0.018±0.039	84.8	1114
	July	3.46 x 10^{-3}±1.28 x 10^{-3}	0.106±0.031		
	August	2.49 x 10^{-3}±1.09 x 10^{-3}	0.050±0.051		
Phenylethyl alcohol	June	4.31 x 10^{-3}±2.36 x 10^{-3}	0.047±0.053	90.8	1127
	July	9.37 x 10^{-4}±1.72 x 10^{-4}	0.156±0.016		
	August	9.47 x 10^{-4}±3.93 x 10^{-4}	0.093±0.049		
2(10)-Pinen-3-ol	June	2.19 x 10^{-2}±4.78 x 10^{-3}	0.092±0.021	82.0	1145
	July	1.01 x 10^{-2}±2.14 x 10^{-3}	0.159±0.018		
	August	3.50 x 10^{-3}±1.25 x 10^{-3}	0.197±0.042		
Pinocarveol	June	2.46 x 10^{-2}±7.05 x 10^{-3}	0.078±0.028	86.0	1153
	July	1.01 x 10^{-2}±2.16 x 10^{-3}	0.159±0.018		
	August	1.22 x 10^{-4}±5.04 x 10^{-5}	0.063±0.048		
Camphor	June	3.11 x 10^{-3}±1.22 x 10^{-3}	0.099±0.038	95.0	1159
	July	5.61 x 10^{-4}±4.46 x 10^{-4}	0.167±0.053		
	August	4.87 x 10^{-4}±1.67 x 10^{-4}	0.196±0.040		

Compound	Month	E_s	β	MF	RI
Pinocarvone	June	$2.62 \times 10^{-2} \pm 7.19 \times 10^{-3}$	0.084±0.026	77.5	1171
	July	$5.00 \times 10^{-4} \pm 3.14 \times 10^{-4}$	0.178±0.052		
	August	$1.93 \times 10^{-4} \pm 8.51 \times 10^{-5}$	0.239±0.054		
Borneol	June	$1.13 \times 10^{-3} \pm 7.14 \times 10^{-4}$	0.032±0.061	-	1185
	July	$6.42 \times 10^{-4} \pm 1.41 \times 10^{-4}$	0.141±0.019		
	August	$1.80 \times 10^{-4} \pm 5.37 \times 10^{-5}$	0.179±0.035		
Terpinene-4-ol	June	$5.86 \times 10^{-3} \pm 2.02 \times 10^{-3}$	0.107±0.033	95.7	1188
	July	$3.80 \times 10^{-5} \pm 3.93 \times 10^{-5}$	0.126±0.084		
	August	$2.65 \times 10^{-5} \pm 1.56 \times 10^{-5}$	-0.006±0.061		
1-Methylphenyl-ethanone	June	$8.31 \times 10^{-4} \pm 7.02 \times 10^{-4}$	0.119±0.082	90.5	1190
	July	$2.18 \times 10^{-4} \pm 9.93 \times 10^{-5}$	0.189±0.038		
	August	$1.36 \times 10^{-4} \pm 6.82 \times 10^{-5}$	0.137±0.054		
Myrtenal	June	$1.12 \times 10^{-2} \pm 3.70 \times 10^{-3}$	0.091±0.032	89.0	1199
	July	$4.79 \times 10^{-3} \pm 1.13E \times 10^{-3}$	0.154±0.020		
	August	$5.65 \times 10^{-4} \pm 5.17 \times 10^{-4}$	0.249±0.107		
Myrtenol	June	$1.09 \times 10^{-2} \pm 5.88 \times 10^{-3}$	0.106±0.052	92.1	1209
	July	$2.54 \times 10^{-3} \pm 7.45 \times 10^{-4}$	0.112±0.025		
	August	$9.10 \times 10^{-4} \pm 2.41 \times 10^{-4}$	0.144±0.031		
α-Fenchyl acetate	June	$1.02 \times 10^{-2} \pm 5.88 \times 10^{-3}$	0.077±0.037	94.3	1218
	July	$2.53 \times 10^{-4} \pm 6.89 \times 10^{-5}$	0.186±0.024		
	August	$1.17 \times 10^{-4} \pm 3.55 \times 10^{-5}$	0.139±0.035		

Compound	Month	E_s	β	MF	RI
Verbenone	June	$1.84 \times 10^{-2} \pm 6.97 \times 10^{-3}$	0.105±0.037	73.9	1222
	July	$5.64 \times 10^{-3} \pm 1.62 \times 10^{-3}$	0.115±0.024		
	August	$8.77 \times 10^{-6} \pm 2.82 \times 10^{-5}$	0.361±0.392		
Trans-Carveol	June	$1.41 \times 10^{-2} \pm 5.57 \times 10^{-3}$	0.087±0.038	89.6	1235
	July	$1.75 \times 10^{-4} \pm 9.94 \times 10^{-5}$	0.291±0.050		
	August	$1.34 \times 10^{-5} \pm 1.16 \times 10^{-5}$	0.482±0.107		
l-Carvone	June	$2.00 \times 10^{-3} \pm 2.13 \times 10^{-3}$	0.131±0.103	95.0	1252
	July	$4.48 \times 10^{-4} \pm 6.88 \times 10^{-5}$	0.113±0.013		
	August	$3.08 \times 10^{-4} \pm 1.30 \times 10^{-4}$	0.190±0.050		
Piperitone	June	$8.37 \times 10^{-4} \pm 9.13 \times 10^{-4}$	0.015±0.105	90.5	1262
	July	$1.06 \times 10^{-4} \pm 4.89 \times 10^{-5}$	0.107±0.039		
	August	$1.36 \times 10^{-4} \pm 5.54 \times 10^{-5}$	0.170±0.048		
Isopiperitenone	June	$2.62 \times 10^{-3} \pm 2.05 \times 10^{-3}$	0.174±0.075	89.9	1282
	July	$5.78 \times 10^{-3} \pm 1.56 \times 10^{-3}$	0.123±0.023		
	August	$1.68 \times 10^{-3} \pm 5.06 \times 10^{-4}$	0.193±0.035		
Benzenepropanoic acid methyl ester	June	$2.11 \times 10^{-3} \pm 3.37 \times 10^{-4}$	0.011±0.015	93.3	1285
	July	$7.45 \times 10^{-4} \pm 1.36 \times 10^{-4}$	0.068±0.015		
	August	$3.06 \times 10^{-4} \pm 1.56 \times 10^{-4}$	0.147±0.060		

Compound	Month	E_s	β	MF	RI
Unknown Sesquiterpene I	June	$1.15 \times 10^{-2} \pm 1.24 \times 10^{-2}$	0.055±0.104	90.5	1293
	July	$1.00 \times 10^{-2} \pm 4.44 \times 10^{-3}$	0.118±0.037		
	August	$5.77 \times 10^{-5} \pm 3.15 \times 10^{-5}$	0.232±0.064		
Thymol	June	$2.85 \times 10^{-3} \pm 8.92 \times 10^{-4}$	0.104±0.030	81.6	1309
	July	$8.95 \times 10^{-4} \pm 2.65 \times 10^{-4}$	0.185±0.025		
	August	$8.45 \times 10^{-4} \pm 2.66 \times 10^{-4}$	0.199±0.037		
Myrtenyl acetate	June	$4.81 \times 10^{-3} \pm 1.83 \times 10^{-3}$	0.105±0.037	90.1	1328
	July	$1.57 \times 10^{-3} \pm 3.62 \times 10^{-4}$	0.151±0.019		
	August	$5.85 \times 10^{-5} \pm 3.61 \times 10^{-5}$	0.270±0.072		
Piperitenone	June	$9.55 \times 10^{-4} \pm 5.95 \times 10^{-4}$	0.064±0.060	85.4	1343
	July	$3.68 \times 10^{-4} \pm 9.87 \times 10^{-5}$	0.125±0.023		
	August	$2.50 \times 10^{-4} \pm 8.31 \times 10^{-5}$	0.189±0.039		
Carvyl Acetate	June	$1.56 \times 10^{-3} \pm 5.70 \times 10^{-4}$	0.103±0.035	95.3	1361
	July	$3.55 \times 10^{-4} \pm 8.53 \times 10^{-5}$	0.175±0.020		
	August	$2.41 \times 10^{-4} \pm 8.61 \times 10^{-5}$	0.198±0.042		
Cis-p-Mentha-6,8-dien-2-ol acetate	June	$5.78 \times 10^{-3} \pm 1.72 \times 10^{-3}$	0.068±0.029	84.2	1363
	July	$2.40 \times 10^{-3} \pm 6.88 \times 10^{-4}$	0.120±0.024		
	August	$8.01 \times 10^{-4} \pm 3.01 \times 10^{-4}$	0.199±0.044		
β-Cubebene	June	$1.11 \times 10^{-3} \pm 5.15 \times 10^{-4}$	0.152±0.045	94.7	1388
	July	$5.94 \times 10^{-4} \pm 1.58 \times 10^{-4}$	0.156±0.023		
	August	$1.04 \times 10^{-4} \pm 4.11 \times 10^{-5}$	0.209±0.046		

Compound	Month	E_s	β	MF	RI
Dihydro-α-Ionone	June	$3.25 \times 10^{-4} \pm 2.78 \times 10^{-4}$	0.061±0.078	83.2	1413
	July	$6.63 \times 10^{-4} \pm 4.00 \times 10^{-4}$	0.103±0.051		
	August	$3.24 \times 10^{-4} \pm 2.77 \times 10^{-4}$	0.137±0.100		
Trans-Caryophyllene	June	$2.20 \times 10^{-3} \pm 1.23 \times 10^{-3}$	0.066±0.054	76.7	1418
	July	$6.64 \times 10^{-4} \pm 1.99 \times 10^{-4}$	0.179±0.025		
	August	$2.90 \times 10^{-4} \pm 1.09 \times 10^{-4}$	0.195±0.044		
Germacrene	June	$2.96 \times 10^{-4} \pm 1.17 \times 10^{-4}$	0.172±0.038	86.8	1428
	July	$1.30 \times 10^{-4} \pm 5.33 \times 10^{-5}$	0.163±0.035		
	August	$5.86 \times 10^{-5} \pm 3.46 \times 10^{-5}$	0.155±0.069		
Unknown Sesquiterpene II	June	$5.65 \times 10^{-4} \pm 6.00 \times 10^{-4}$	0.272±0.103	91.5	1439
	July	$3.90 \times 10^{-4} \pm 3.00 \times 10^{-4}$	0.222±0.065		
	August	$2.22 \times 10^{-4} \pm 8.24 \times 10^{-5}$	0.224±0.044		
β-Chamigrene	June	$9.23 \times 10^{-5} \pm 1.15 \times 10^{-4}$	0.291±0.120	74.4	1441
	July	$1.08 \times 10^{-4} \pm 8.86 \times 10^{-5}$	0.183±0.072		
	August	$5.74 \times 10^{-5} \pm 3.21 \times 10^{-5}$	0.187±0.066		
6-Endo-Hydroxyborneol	June	$1.13 \times 10^{-3} \pm 3.85 \times 10^{-4}$	0.122±0.033	-	1443
	July	$3.82 \times 10^{-4} \pm 8.72 \times 10^{-5}$	0.168±0.019		
	August	$2.05 \times 10^{-4} \pm 1.23 \times 10^{-4}$	0.224±0.071		

Compound	Month	E_s	β	MF	RI
α-Cubebene	June	$8.52 \times 10^{-4} \pm 6.26 \times 10^{-4}$	0.180±0.071	86.2	1449
	July	$6.38 \times 10^{-4} \pm 2.62 \times 10^{-4}$	0.185±0.035		
	August	$8.22 \times 10^{-4} \pm 5.29 \times 10^{-4}$	0.195±0.076		
Alloaromadendrene	June	$2.92 \times 10^{-3} \pm 2.16 \times 10^{-3}$	0.278±0.072	94.5	1462
	July	$1.84 \times 10^{-3} \pm 1.39 \times 10^{-3}$	0.212±0.064		
	August	$1.19 \times 10^{-4} \pm 6.39 \times 10^{-5}$	0.198±0.063		
α-Amorphene	June	$2.34 \times 10^{-3} \pm 7.95 \times 10^{-4}$	0.162±0.033	95.5	1475
	July	$1.27 \times 10^{-3} \pm 4.19 \times 10^{-4}$	0.142±0.028		
	August	$3.84 \times 10^{-4} \pm 1.63 \times 10^{-4}$	0.220±0.050		
Ledene	June	$7.28 \times 10^{-4} \pm 5.53 \times 10^{-4}$	0.205±0.073	85.6	1493
	July	$2.43 \times 10^{-4} \pm 1.35 \times 10^{-4}$	0.203±0.047		
	August	$5.91 \times 10^{-5} \pm 3.01 \times 10^{-5}$	0.199±0.060		
γ-Selinene	June	$6.79 \times 10^{-4} \pm 4.30 \times 10^{-4}$	0.131±0.061	82.0	1496
	July	$2.43 \times 10^{-4} \pm 1.53 \times 10^{-4}$	0.145±0.053		
	August	$5.07 \times 10^{-5} \pm 3.12 \times 10^{-5}$	0.225±0.072		
α-Muurolene	June	$9.84 \times 10^{-4} \pm 3.98 \times 10^{-4}$	0.184±0.039	97.0	1498
	July	$5.30 \times 10^{-4} \pm 2.18 \times 10^{-4}$	0.161±0.035		
	August	$1.78 \times 10^{-4} \pm 7.43 \times 10^{-5}$	0.237±0.049		
α-Farnesene	June	$1.27 \times 10^{-4} \pm 6.55 \times 10^{-5}$	0.223±0.050	92.3	1507
	July	$1.42 \times 10^{-4} \pm 7.32 \times 10^{-5}$	0.178±0.044		
	August	$5.53 \times 10^{-5} \pm 2.93 \times 10^{-5}$	0.233±0.066		

Compound	Month	E_s	β	MF	RI
δ-Cadinene	June	2.20 x 10^{-3}±1.19 x 10^{-3}	0.213±0.053	95.1	1519
	July	1.43 x 10^{-3}±6.15 x 10^{-4}	0.179±0.036		
	August	4.52 x 10^{-4}±2.00 x 10^{-4}	0.236±0.052		
Unknown Sesquiterpene III	June	1.01 x 10^{-3}±3.04 x 10^{-4}	0.107±0.029	94.4	1532
	July	8.42 x 10^{-4}±3.79 x 10^{-4}	0.171±0.038		
	August	4.33 x 10^{-4}±1.79 x 10^{-4}	0.196±0.048		
Cadina-1,2,4-diene	June	9.16 x 10^{-4}±5.83 x 10^{-4}	0.165±0.061	93.2	1540
	July	6.61 x 10^{-4}±2.47 x 10^{-4}	0.174±0.032		
	August	2.3 x 10^{-4}±7.52 x 10^{-5}	0.222±0.037		
Palustrol	June	1.22 x 10^{-3}±6.15 x 10^{-4}	0.221±0.049	96.0	1568
	July	4.36 x 10^{-4}±3.24 x 10^{-4}	0.217±0.065		
	August	2.27 x 10^{-5}±7.40 x 10^{-5}	0.279±0.354		
Spathulenol	June	3.90 x 10^{-4}±2.23 x 10^{-4}	0.234±0.055	88.4	1576
	July	1.06 x 10^{-4}±8.64 x 10^{-5}	0.288±0.069		
	August	1.08 x 10^{-4}±5.07 x 10^{-5}	0.231±0.055		
Aromadendran	June	1.41 x 10^{-4}±3.70 x 10^{-5}	0.160±0.025	85.3	1579
	July	5.65 x 10^{-5}±1.25 x 10^{-5}	0.164±0.019		
	August	2.66 x 10^{-5}±6.97 x 10^{-6}	0.204±0.031		

Compound	Month	E_s	β	MF	RI
Viridiflorol	June	$3.15 \times 10^{-3} \pm 2.76 \times 10^{-3}$	0.367±0.085	93.1	1598
	July	$1.51 \times 10^{-3} \pm 4.97 \times 10^{-4}$	0.133±0.028		
	August	$1.78 \times 10^{-3} \pm 6.16 \times 10^{-4}$	0.180±0.041		
δ-Cadinol	June	$4.66 \times 10^{-3} \pm 2.37 \times 10^{-3}$	-0.005±0.049	84.6	1665
	July	$1.10 \times 10^{-3} \pm 5.17 \times 10^{-4}$	0.133±0.040		
	August	$9.85 \times 10^{-4} \pm 4.57 \times 10^{-4}$	0.169±0.055		
Cadalene	June	$8.01 \times 10^{-4} \pm 3.30 \times 10^{-4}$	0.201±0.040	93.1	1670
	July	$4.19 \times 10^{-4} \pm 1.58 \times 10^{-4}$	0.196±0.032		
	August	$4.08 \times 10^{-4} \pm 1.37 \times 10^{-4}$	0.198±0.040		
Benzyl benzoate	June	$7.44 \times 10^{-5} \pm 2.37 \times 10^{-5}$	0.195±0.031	94.5	1768
	July	$5.86 \times 10^{-6} \pm 2.49 \times 10^{-6}$	0.294±0.038		
	August	$7.49 \times 10^{-6} \pm 5.57 \times 10^{-6}$	0.379±0.087		
5-β-Androstane	June	$2.84 \times 10^{-5} \pm 3.89 \times 10^{-5}$	0.096±0.132	80.1	1926
	July	$4.50 \times 10^{-5} \pm 1.5 \times 10^{-5}$	0.136±0.029		
	August	$5.00 \times 10^{-5} \pm 1.76 \times 10^{-5}$	0.152±0.041		
Manoyl oxide	June	$1.32 \times 10^{-5} \pm 7.46 \times 10^{-6}$	0.122±0.056	92.1	1933
	July	$1.83 \times 10^{-5} \pm 4.25 \times 10^{-6}$	0.108±0.020		
	August	$2.14 \times 10^{-5} \pm 8.25 \times 10^{-6}$	0.139±0.045		
Ent-16-kaurene	June	$3.13 \times 10^{-6} \pm 4.60 \times 10^{-6}$	0.089±0.142	96.3	1941
	July	$3.31 \times 10^{-6} \pm 8.33 \times 10^{-7}$	0.066±0021		
	August	$2.84 \times 10^{-6} \pm 1.16 \times 10^{-6}$	0.119±0.048		

Compound	Month	E_s	β	MF	RI
Verticillol	June	$3.23 \times 10^{-6} \pm 3.03 \times 10^{-6}$	0.079±0.093	75.1	1947
	July	$2.20 \times 10^{-5} \pm 5.49 \times 10^{-6}$	0.063±0.021		
	August	$1.64 \times 10^{-5} \pm 7.69 \times 10^{-6}$	0.130±0.055		
Cembrene	June	$2.28 \times 10^{-5} \pm 1.80 \times 10^{-5}$	0.124±0.076	81.5	1944
	July	$1.21 \times 10^{-5} \pm 4.40 \times 10^{-6}$	0.103±0.031		
	August	$1.13 \times 10^{-5} \pm 7.17 \times 10^{-6}$	0.131±0.075		
5-α-Androstan-6-one	June	$7.55 \times 10^{-7} \pm 1.06 \times 10^{-6}$	0.205±0.134	73.2	1984
	July	$3.32 \times 10^{-6} \pm 9.45 \times 10^{-7}$	0.159±0.024		
	August	$3.57 \times 10^{-6} \pm 1.17 \times 10^{-6}$	0.152±0.038		
